# Literature-based human milk nutrient composition values for use in North American food composition databases

**DOI:** 10.1016/j.ajcnut.2026.101252

**Published:** 2026-04-07

**Authors:** Kathryn E Hopperton, Samadhi Thavarajah, Jaspreet Ahuja, Kellie Casavale, Subhadeep Chakrabarti, Kimberlea Gibbs, Tina Irrer, Stephanie K Nishi, Sophie Parnel, Pamela Pehrsson, Melanie Stanton, Dennis Anderson-Villaluz, Krista A Zanetti, Ashley J Vargas

**Affiliations:** 1 Nutrition Research Division, Bureau of Nutritional Sciences, Food and Nutrition Directorate, Health Canada, Ottawa, ON, Canada; 2 National Institute of Environmental Health Sciences, NIH, Durham, NC, United States; 3 USDA, Agriculture Research Service, Beltsville Human Nutrition Research Center, Beltsville, MD, United States; 4 Office of Surveillance Strategy and Risk Prioritization, Human Foods Program, Food and Drug Administration, United States Department of Health and Human Services, College Park, MD, United States; 5 Nutrition Premarket Assessment Division, Bureau of Nutritional Sciences, Food and Nutrition Directorate, Health Canada, Ottawa, ON, Canada; 6 National Institute of Diabetes and Digestive and Kidney Diseases, NIH, Bethesda, MD, United States; 7 Office of Disease Prevention and Health Promotion, United States Department of Health and Human Services, Rockville, MD, United States; 8 Office of Nutrition Research, Office of the Director, NIH, Bethesda, MD, United States

**Keywords:** human milk, human milk nutrients, breast milk, nutrient reference data, human milk composition, food composition databases, fatty acids, vitamins, minerals, macronutrients

## Abstract

**Background:**

The profile for human milk (HM) nutrient composition jointly used by Canada and the United States [Standard Reference (SR), legacy] is largely based on >40-y-old studies. In 2018, it was deemed unsuitable by the United States Department of Agriculture for estimating current nutrient exposures.

**Objectives:**

To review data available in the literature to develop interim HM nutrient profile data values.

**Methods:**

Two reviewers screened and extracted data from 3 recent systematic reviews covering the period from 1980‒2022 and 1 large Canadian biomonitoring study. Eligible studies: reported quantitative concentrations of ≥1 component of interest (energy, macronutrients, vitamins, minerals, or fatty acids), included data specific to United States or Canadian participants, reported on mature milk (>21 d postpartum) for term-born infants (>37 wk of gestation), had samples collected within the first 6 mo of lactation, and used appropriate methods for milk collection and nutrient analysis. Studies were combined as weighted means and pooled standard deviations and compared with the existing SR and international literature.

**Results:**

Updated data were identified for >40 HM components, including some that had previously been assigned 0 in SR legacy (e.g., DHA), or estimated from other foods (e.g., vitamin K). Concentrations for interim HM nutrient profile data differed by >20% from values in the SR legacy for total fat, iron, manganese, and most vitamins and fatty acids. Eligible data were lacking for niacin, vitamin B-12, vitamin C, and total vitamin D, and were only available from single studies for manganese, β-carotene, thiamin, riboflavin, pantothenic acid, vitamin B6, total choline, 25-hydroxyvitamin D, vitamin E, cholesterol, and some fatty acids.

**Conclusions:**

These findings summarize data available to develop an interim nutrient profile for North American HM that could be used until an empirically measured profile can be developed. They also highlight gaps in the literature to be addressed by future studies.

## Introduction

Human milk (HM) is recommended as the sole source of nutrition for infants to 6 mo of age, and as a complementary component of the diet for older infants and young children up to age 2 y and beyond [[1], [2], [3]]. HM composition (HMC) data are used for the development of Dietary Reference Intakes (DRIs) and dietary guidance for infancy and lactation, the development and regulation of infant formulas, and for estimating nutrient and chemical exposures of infants and young children [4]. Despite its importance, current nationally representative HMC data are lacking in the United States and Canada. The profile for HMC that is used by the USDA FoodData Central and the Canadian Nutrient File has not been updated for most nutrients in almost 50 y, and in 2018, was moved to legacy status by the USDA, meaning that it can no longer be used to support federal policies and programs [5,6]. Since then, reviews of North American HMC literature have noted limitations in the available published data, including a lack of detail or representativeness of the populations used, small sample sizes, and the use of methods for the collection, storage, or analysis of HM that do not align with current best practices [7,8]. Similar issues were noted for HMC data from other high and upper-middle–income countries in a recent National Academies of Sciences, Engineering, and Medicine (NASEM) evidence scan that assessed the availability of data to support a potential update to the DRIs [9].

The HM Composition Initiative (HMCI) was established in 2018 as a joint undertaking between the United States and Canadian federal agencies to foster efforts to encourage, coordinate, and improve HMC data collection in the 2 countries [4,10]. Recognizing the limitations of existing data sources, the HMCI recognized the need for a new, representative study of North American HMC using appropriate collection and analytical methods [4]. Until such time that this study can be conducted, the HMCI has undertaken a process to determine what data is available to develop an interim HM nutrient profile based on updated, available North American literature data, combined with recently analyzed national Canadian biomonitoring data [interim HM nutrient profile data (iHMNutD)]. The objective of this paper is to describe the data available.

## Methods

### Data sources

This work is a literature synthesis of eligible North American data from 3 previously published systematic reviews and 1 national biomonitoring study [[7], [8], [9], [11]]. These data sources were selected by the coauthors in alignment with policy needs for updating federal food composition databases.

The first review, conducted by the USDA and published in 2018 [7], covered studies published between 1980 and 2017, whereas a second, commissioned by the HMCI and published by Mohr et al. [8] in 2023, covered studies for the period from 2017 to 2022. The scope of these reviews was mature term HM from participants living in Canada and the United States. These reviews did not exclude studies on the basis of HM collection and analytical methods, though the Mohr et al. [8] review extracted and commented on this data using the same criteria as applied for iHMNutD (described below). The third source review was a 2020 NASEM evidence scan, which covered the period from 1980 to 2020 and included studies using eligible methods similar to those in Supplemental Table 1 to measure nutrients in term HM from participants living in high or middle-high-income countries [9]. Details of the search strategies of these reviews have been published previously [[7], [8], [9]].

National biomonitoring data on HM minerals from the Canadian Maternal-Infant Research on Environmental Chemicals study were published after 2022 [11]. These data were also included in the development of iHMNutD by consensus of the study team, as it is the largest study of HM minerals in North America published to date, and a sub-analysis of participants meeting the inclusion criteria of iHMNutD was available [11].

### Inclusion criteria

For inclusion in iHMNutD, studies had to: *1*) Provide quantitative data for ≥1 macronutrient, vitamin, mineral, or fatty acid currently included in North American food composition databases (see Supplemental Table 1 for full list) per mass or volume of HM, *2*) be published after 1 January, 1980, *3*) include data specifically for participants residing in the United States or Canada (in studies with participants from multiple countries, results must be reported separately for United States or Canadian participants), *4*) provide data for mature milk (>21 d postpartum) for term-born infants (>37 wk of gestation), *5*) have samples collected within the first year of lactation (separately for >21 d-6 mo or 7‒12 mo postpartum), and *6*) have appropriate methods for HM collection, and nutrient analysis, as summarized in Supplemental Table 1 (adapted from [8,9]). The eligible collection and analysis methods vary by component because different methods are recommended for different nutrients, and can substantially affect the validity of nutrient content estimates [4,12].

As food composition databases are used to estimate current nutrient exposures in North America and not to produce recommended intakes, studies were not excluded solely on the basis of characteristics such as multiple births, parity, maternal age, maternal health status [e.g., BMI (in kg/m^2^), chronic disease], or maternal health behaviors (e.g., smoking, over the counter supplement use) that occur in the general population. However, when these characteristics were the focus of the study (e.g., randomized controlled trial of a nutritional supplement, or a comparison of HMC between participants with and without obesity), data from the control group were used.

### Data extraction and conversion

The following data points were manually extracted from each systematic review or the original studies as applicable: first author, year, location, timing of sample collection postpartum, sample size, analytical method, milk sampling type, milk collection time, nutrient central tendency, and nutrient variance. Central tendency and variance data were converted to match the units in the legacy USDA Standard Reference (SR) legacy for HM and the Canadian Nutrient File food composition databases, which were in mass units (gram, milligram, microgram, nanogram) per 100 g of HM [5,6]. The HM specific gravity of 1.031 g/mL was used to convert units per milliliter to units per gram [7]. When SE, IQR, or 95% confidence intervals were reported, relevant equations from the Cochrane handbook methods were used to convert to SD [13]. Where data from multiple time points were available within a single study, data from >21 d to <7 mo and 7 mo to 12 mo were combined as weighted means. Screens for eligibility, data extraction, and unit conversions were completed by 1 author and validated by a second, with any discrepancies resolved by consensus.

Studies on other components currently listed in the SR legacy database, including individual amino acids, caffeine, alcohol, theobromine, lycopene, lutein, ash, and fiber, were screened but excluded because they were not part of the search strategies for all the reviews that served as data sources.

### Statistical analysis

Summary statistics were calculated only for the 0‒6 mo postpartum time point due to the limited availability of data points from 7‒12 mo. Statistical analyses were performed using STATA standard edition version 18.0. Studies were weighted by sample size using Equation 1 below, where μ represents the mean and *n*, the sample size for each study. Pooled SDs were calculated using Equation 2*,* accounting for differences in sample size. Equation 3 was used to calculate the fold differences from comparators. Differences within 0.2-fold (20%), i.e., 0.8–1.2, were considered to be within the regular range of expected analytical variability for food composition data.

(1) $$\frac{\sum \left(\mu_{1}\times n_{1}\right)\ldots \left(\mu_{x}\times n_{x}\right)}{\sum \left(n_{1}-n_{x}\right)}$$

(2) $$\sqrt{\frac{\left(n_{1}-1\right){{SD}_{1}}^{2}+\left(n_{2}-1\right){{SD}_{2}}^{2}\ldots +\left(n_{x}-1\right){{SD}_{x}}^{2}}{n_{1+}n_{2+}\ldots n_{x}-n_{x}}}$$

(3) $$\left(\frac{Comparator}{iHMNutD}\right)$$

For fatty acids, values for totals were calculated as sums and pooled variance from other individual fatty acids reported in the papers identified: SFA (sum of 4:0, 6:0, 8:0, 10:0, 12:0, 14:0, 16:0, and 18:0), MUFAs (sum of total 16:1, total 18:1, 20:1, 22:1), n‒3 PUFAs [sum of 18:3n‒3 (α-linolenic acid, ALA), 20:3n‒3, 22:5n‒3, 20:5n‒3 (EPA), 22:6n‒3 (docosahexaenoic acid, DHA)], n‒6 PUFA [sum of 18:2n‒6 (linoleic acid), 20:4n‒6 (arachidonic acid), and 22:4n‒6]. Fatty acids are more commonly reported in percent composition in the literature, as opposed to quantitative values, which were required by our inclusion criteria. We therefore converted our iHMNutD fatty acid values to percent composition using the iHMNutD total lipid value (3.53 g/100 g) converted to total fatty acids using a conversion factor of 0.945 as recommended by FAO for milk and milk products [14] to facilitate literature comparisons using Equation 4.

(4) $$\left(\frac{iHMNut\;fatty\;acid}{iHMNut\;total\;fatty\;acids}\right)\times 100\%)$$

### Comparison with other literature

To provide a point of comparison for values obtained in iHMNutD, we calculated weighted means and pooled SDs as described above, based on comparable studies identified in the NASEM evidence scan, for all available nutrients with iHMNutD values [9]. The NASEM review used the same HM collection and analysis criteria as iHMNutD but differed in other respects, notably by including studies from high- and upper-middle income countries outside North America, which increased the number of studies available for most nutrients. This review also differed from the iHMNutD process in that it included studies of milk collected <1-mo postpartum, and excluded studies based on maternal age <18, maternal and infant general health, and multiple births. As these NASEM review values were generated only for comparison purposes, extractions and calculations were conducted by a single reviewer.

The NASEM evidence scan did not include energy, and identified no eligible studies for total vitamin D. For these nutrients, we instead compared iHMNutD with values from a 2014 systematic review by Gidrewicz and Fenton [15], which included studies from North America, Europe, Australia, Israel, and Japan, and North American values from a 2023 systematic review by Rios-Leyvraz and Yao [16], respectively.

Cholesterol and most individual fatty acids reported in food composition databases were also not available in the NASEM evidence scan. We therefore compared these nutrients with values from a recent large systematic review of global HM lipid composition [17]. HM fatty acid composition is known to vary substantially with diet and supplementation. Therefore, we compared iHMNutD values with data from a 2019 Canadian study of HM fatty acid composition, which included >1000 participants and is likely more representative of the North American population [18].

While this manuscript was under review, the Mothers, Infants and Lactation Quality (MILQ) studies were published, which included reference values for macronutrients and select vitamins and minerals in HM collected through the first 8.5 mo of lactation for over 1200 women in Bangladesh, Brazil, Denmark, and the Gambia [[19], [20], [21], [22]]. Given the size and importance of this study to estimates of dietary requirements, we also compared iHMNutD to the 1–6-mo medians from MILQ where available. MILQ was designed to provide estimates of healthy HM nutrient composition that could be used to set human dietary requirements, whereas iHMNutD was designed to estimate actual North American exposures via HM. MILQ therefore recruited participants from 4 diverse countries, whereas iHMNutD was restricted to data from participants in Canada and the United States. MILQ also applied inclusion criteria that were not used in iHMNutD, including restrictions based on participant characteristics such as maternal age, BMI, past or current medical problems, alcohol, smoking, diet type (excluding vegan or macrobiotic diets), micronutrient supplementation (iron and folic acid permitted in all sites, and vitamin D and calcium in Bangladesh and Denmark), intake of fortified foods (except iodized salt), multiple gestations, dietary diversity, infant birth weight and congenital anomalies [23]. Macronutrients in MILQ were analyzed by near-infrared spectroscopy, which did not meet the methodological inclusion criteria of iHMNutD, although other analytical methods did meet our inclusion criteria.

Nutrient composition values were not expected to be identical between iHMNutD and these other data sources due to the differences described above. Rather, comparisons were made to provide a range of plausible literature values to guide decisions about which values to incorporate into food composition databases, particularly when iHMNutD values were based on a small number of participants or studies.

### Subject matter expert review

An early draft of iHMNutD values was reviewed by 4 to 5 experts per nutrient recruited from the Academic Volunteer Interest Expert Group, a standing group of academic HM researchers assembled by the HMCI. All reviewers hold current appointments at United States or Canadian academic institutions and publish regularly in the field of HM and lactation. Experts were provided a comprehensive summary of the project, methods, data sources, and draft data, and were asked to comment on the feasibility of the project, the analytical and sampling inclusion criteria, the data extracted, and if they had any suggestions, additional data flags, or other information that could aid in appropriate use and interpretation of each nutrient’s findings. Based on these reviews, the project was refined, and data flags were proposed to highlight nutrients for which iHMNutD was based on a single study, a small number of participants (<20), and/or data published entirely before the year 2000. The intention of these flags was to provide updated values, but also to clearly communicate uncertainty to data users.

## Results

### Overview of studies identified

**FIGURE 1:**
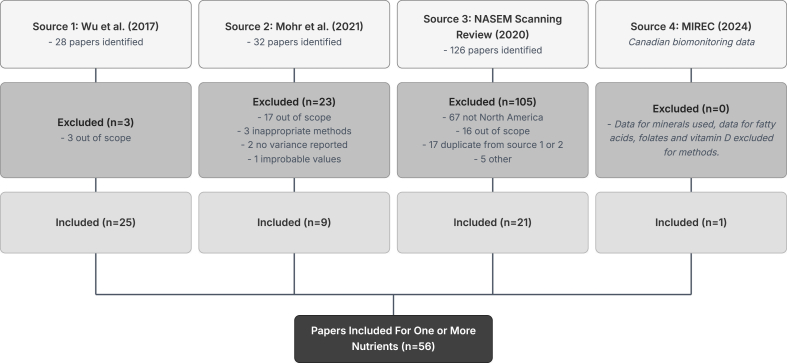
Flow diagram for the identification of articles for iHMNutD^1^. ^1^Flow diagram showing the selection of studies for inclusion in iHMNutD from 3 source reviews: source 1—Wu et al. [7], source 2—Mohr et al. [8], and source 3—NASEM scanning review [9] and 1 Canadian biomonitoring study published after the reference period of the other source reviews: source 4—MIREC [11]. Details of included studies are provided in Supplemental Table 2. The main reasons for exclusion of papers were that they were not from North America, or that they were out of scope, either because they provided values for elements not currently included in iHMNutD (e.g., human milk oligosaccharides, amino acids, and milk volume), or because they provided concentrations for time periods <3 wk or >6 mo postpartum. Other reasons for exclusion included a lack of reporting of variance, data from RCTs of nutrient supplements for which composition values were not provided for the placebo group, analytical methods not included in Supplemental Table 1, composition values not being reported per volume or mass of human milk, and in 1 case, values considered implausible upon subject matter expert review (total fat concentration >30-fold lower than established ranges for human milk). iHMNutD, interim human milk nutrient profile data; MIREC, Maternal-Infant Research on Environmental Chemicals; NASEM, National Academies of Sciences, Engineering, and Medicine; RCT, randomized controlled trial.

Overall, 28 studies from the Wu et al. [7] review, 32 from the Mohr et al. [8] review, and 126 from the NASEM evidence scan were evaluated for inclusion in iHMNutD, along with Canadian biomonitoring data from the Canadian Maternal-Infant Research on Environmental Chemicals pregnancy cohort [11]. Of these, 25 papers from Wu et al. [7], 9 papers from Mohr et al. [8], and 38 papers from the NASEM evidence scan reported 1 or more nutrients for the 0–6-month time period among North American participants. Upon exclusion of duplicates, this left a total of 56 eligible articles (Figure 1 [[7], [8], [9],^11^]). Details of the included studies are shown in Supplemental Table 2. For the Wu et al. [7] and Mohr et al. [8] reviews, the most common reason for exclusion was that the article reported data for a HM component or time period postpartum that did not fit the inclusion criteria (e.g., HM oligosaccharides, volume, and amino acids), followed by use of a method of HM analysis other than those outlined in Supplemental Table 1. For the NASEM evidence scan, the most common reason for exclusion was that study participants were in non-North American countries. Upon subject matter expert review and consensus of the study team, 1 additional study that otherwise met the inclusion criteria was excluded for having values for total fat >30-fold lower than established ranges for HM [15,24].

Eligible studies were identified for energy, protein, total lipid, lactose, cholesterol, the following minerals: calcium, chloride, copper, iodine, iron, magnesium, manganese, phosphorus, potassium, selenium, sodium, and zinc; the following vitamins and related compounds: vitamin A, β-carotene, thiamin, riboflavin, pantothenic acid, vitamin B-6, folate, water-soluble choline, total choline, 25-hydroxyvitamin D [25(OH)D], vitamin E, and vitamin K and; and 20 fatty acids. The details of all included studies are provided in Supplemental Table 2. No studies were identified for niacin, vitamin B-12, vitamin C, fluoride, or total vitamin D. The number of studies identified for a particular nutrient ranged from 1 (for β-carotene, riboflavin, thiamin, pantothenic acid, vitamin B-6, total choline, 25(OH)D, vitamin E, manganese cholesterol and fatty acids 4:0 and 20:1n‒9) up to 15 (for zinc). The number of participants with available data for each nutrient ranged from 10 (for cholesterol) up to 1080 (for zinc).

#### Comparison with SR legacy

A comparison between iHMNutD summary statistics and the existing mean values in the SR legacy, as well as data flags proposed in subject matter expert review, is shown in Table 1. Values for iHMNutD were within 20% of the corresponding SR legacy value for energy, protein, calcium, copper, magnesium, phosphorus, potassium, selenium, sodium, zinc, pantothenic acid, vitamin B-6, and ALA, but differed by >20% for total lipid, iron, manganese, vitamin A, β-carotene, thiamin, riboflavin, folate, total choline, 25(OH)D, vitamin E, vitamin K, and other fatty acids. For example, the SR legacy value was 0.6-fold lower than the weighted mean of the studies identified in iHMNutD for iron [0.03 mg/100 g compared to 0.05 (SD 0.04) mg/100 g for iHMNutD based on 7 studies including a total of 231 participants] and >130-fold higher than iHMNutD for manganese [0.026 mg/100 g compared to 0.0002 (SD 0.0002) mg/100 g in iHMNutD based on 1 study of 671 participants]. Concentrations were set at 0 in SR legacy for several fatty acids known to be present in HM, including EPA and DHA. iHMNutD provides non-zero values for these and other fatty acids, with study and participant numbers ranging from 1 study of 19 participants (for 4:0) up to 5 studies including a total of 145 participants for DHA.

**TABLE 1:** Interim human milk nutrient profile data summary statistics and comparison to USDA standard reference and comparable studies for macronutrients, minerals, vitamins, and fatty acids^1^

USDA standard reference legacy		Unit (per 100 g)	iHMNutD				Fold difference from SR legacy	Data flags
Nutrient	Value		*n* studies	Sample size	Weighted mean	Pooled SD		
Energy	70	kcal	9	208	67.44	9.16	1.04	Before 2000^4^
Metabolizable energy^2^			4	108	69.49	9.69	1.01	
Macronutrients								
Protein^3^	1.03	g	8	199	0.93	0.14	1.11	Before 2000^4^
Total lipid	4.38	g	14	350	3.53	1.09	1.24**^^^**	—
Lactose	—	g	4	106	6.79	1.06	—	—
Minerals								
Calcium	32	mg	5	841	31.03	5.19	1.03	—
Chloride	—	mg	5	139	40.35	11.67	—	Before 2000^4^
Copper	0.052	mg	9	907	0.044	0.020	1.18	—
Iodine	—	*μ*g	2	765	18.25	13.80	—	—
Iron	0.03	mg	7	231	0.050	0.038	0.60**^^^**	—
Magnesium	3	mg	5	841	3.42	0.73	0.88	—
Manganese	0.026	mg	1	671	0.00019	0.00017	136.84**^^^**	1 study^5^
Phosphorus	14	mg	2	765	14.01	2.53	1.00	—
Potassium	51	mg	10	905	55.56	7.31	0.92	—
Selenium	1.8	*μ*g	2	671	1.98	0.48	0.91	—
Sodium	17	mg	12	924	14.73	6.99	1.15	—
Zinc	0.17	mg	15	1080	0.19	0.09	0.90	—
Vitamins								
Vitamin A (retinol)	60	*μ*g	3	46	36.09	3.59	1.66**^^^**	—
β-carotene	7	*μ*g	1	13	2.84	0.11	2.46**^^^**	1 study^5^
Thiamin	0.014	mg	1	13	0.026	0.004	0.53**^^^**	1 study^5^, <20 participants^6^
Riboflavin	0.036	mg	1	13	0.0010	0.0001	**3.72^^^**	1 study^5^, <20 participants^6^
Pantothenic acid	0.223	mg	1	26	0.25	0.07	0.90	Before 2000^4^, 1 study^5^
Vitamin B-6	0.011	mg	1	30	0.012	0.003	0.91	Before 2000^4^, 1 study^5^
Folate	5	*μ*g	5	103	7.46	2.69	0.67**^^^**	—
Water-soluble choline	—	mg	2	321	11.32	3.07	—	—
Total choline	16	mg	1	48	12.10	2.70	1.32**^^^**	1 study^5^
25-hydroxyvitamin D	0.1	*μ*g	1	21	0.13	0.06	0.79**^^^**	1 study^5^
Vitamin E	0.08	mg	1	21	0.37	0.17	0.22**^^^**	1 study^5^
Vitamin K	0.3	*μ*g	3	79	0.19	0.15	1.60**^^^**	—
Fatty acids^7^								
SFA 4:0	0	g	1	19	0.0000	0.0000	Indivisible	1 study^5^, <20 participants^6^
SFA 6:0	0	g	2	48	0.0010	0.0010	Indivisible	—
SFA 8:0	0	g	2	48	0.0034	0.0015	Indivisible	—
SFA 10:0	0.063	g	4	118	0.034	0.010	1.83**^^^**	—
SFA 12:0	0.256	g	4	118	0.17	0.06	1.54**^^^**	—
SFA 14:0	0.321	g	4	118	0.20	0.07	1.58**^^^**	—
SFA 16:0	0.919	g	4	118	0.71	0.09	1.30**^^^**	—
SFA 18:0	0.293	g	4	118	0.23	0.05	1.25**^^^**	—
Total SFA^7^	2.01	g	—	—	1.35	0.14	1.49**^^^**	—
MUFA 16:1	0.129	g	3	99	0.087	0.020	1.48**^^^**	—
MUFA 18:1n‒9	1.48	g	4	118	1.18	0.15	1.25**^^^**	—
MUFA 20:1n‒9	0.04	g	1	29	0.012	0.003	3.33**^^^**	1 study^5^
MUFA 22:1n‒9	0	g	2	48	0.0030	0.0020	Indivisible	—
Total MUFA^8^	1.66	g	—	—	1.28	0.15	1.29**^^^**	—
PUFA 18:3 (ALA)	0.052	g	4	118	0.049	0.015	1.06	—
PUFA 20:3n‒3	—	g	3	58	0.015	0.0094	—	—
PUFA 22:5n‒3	0	g	2	48	0.0042	0.0006	Indivisible	—
PUFA 20:5n‒3 (EPA)	0	g	4	132	0.0023	0.0021	Indivisible	—
PUFA 22:6n‒3 (DHA)	0	g	5	145	0.0066	0.0049	Indivisible	—
n‒3 PUFA^9^	—	g	—	—	0.077	0.019	—	—
PUFA 18:2n‒6 (LA)	0.374	g	4	118	0.56	0.12	0.67**^^^**	—
PUFA 20:4n‒6 (ARA)	0.026	g	4	118	0.014	0.003	1.87**^^^**	—
PUFA 22:4n‒6	—	g	2	48	0.0034	0.002	Indivisible	—
n‒6 PUFA^10^	—	g	—	—	0.58	0.12	—	—
Total PUFA^11^	0.497	g	—	—	0.65	0.12	0.76**^^^**	—
Cholesterol	14	mg	1	10	9.83	0.37	1.42**^^^**	Before 2000^4^, 1 study^5^, <20 participants^6^

Abbreviations: ALA, α-linolenic acid; ARA, arachidonic acid; iHMNutD, interim human milk nutrient profile data; LA, linoleic acid; SR, standard reference.

1 Means and SDs are presented to 2 significant digits for each nutrient in the units found in the SR legacy (where applicable). Fold difference calculated for comparative purposes as SR legacy/iHMNutD. Values within 0.2-fold (20%), i.e., 0.8–1.2, were considered to be within the regular range of expected analytical variability for food composition data. Values indicated by^ are outside this range.

2 Restricted to studies that calculated the human milk energy content from proximates to reflect energy available for metabolism [25].

3 Values for true protein (total nitrogen -non-protein nitrogen) used, with the conversion factor of 6.38 from nitrogen to protein.

4 Proposed data flag indicates where the iHMNutD value is based solely on studies conducted before the year 2000.

5 Proposed data flag indicates where the iHMNutD value is based solely on data from 1 study.

6 Proposed data flag indicates where the iHMNutD value is based on fewer than 20 participants.

7 Total SFA calculated as the sum of weighted mean values in the table for SFA 4:0, 6:0, 8:0, 10:0, 12:0, 14:0, 16:0, and 18:0.

8 Total MUFA calculated as the sum of weighted mean values in the table for MUFA 16:1, 18:1, 20:1, and 22:1.

9 Total n‒3 PUFA calculated as the sum of weighted mean values in the table for ALA, PUFA 20:3n‒3, PUFA 22:5n‒3, EPA, and DHA.

10 Total n‒6 PUFA calculated as the sum of weighted mean values in the table for LA, ARA, and PUFA 22:4n‒6.

11 Total PUFA calculated as the sum of weighted mean values in the table for all n‒6 and n‒6 PUFA.

Direct comparisons with the SR legacy values were not possible for some nutrients. For example, the SR legacy provides data for carbohydrate, whereas studies identified in iHMNutD provided data for total lactose. The NASEM scanning review and the DRI report for carbohydrates use lactose as a proxy for digestible carbohydrate in HM because the majority (>99%) of digestible carbohydrate in HM is lactose [9,26]. The iHMNutD lactose value may therefore be an appropriate alternative for many data applications and is very similar to the current SR legacy value for carbohydrate [6.79 (SD 1.06) mg/100 g for iHMNutD compared to 6.89 mg/100 g in SR legacy]. Similar issues arise for iodine, chloride, and fatty acids 20:3n‒3, 22:4n‒6, total n‒3 PUFA, and total n‒6 PUFA, which were not included in the previous SR legacy values, but were covered in the search strategies of the iHMNutD data sources.

#### Comparison with other literature

Many of the HM composition values generated by the iHMNutD process are within the range of comparable international literature on this topic, including studies identified through the NASEM evidence scan, the MILQ studies, and a global review of HM fatty acid composition. Comparisons are shown between iHMNutD values and other literature for energy, macronutrients, vitamins, and minerals in Table 2, and fatty acids and cholesterol in Table 3.

**TABLE 2:** Comparison of interim human milk nutrient profile data values for macronutrients, minerals, and vitamins with select literature^1^

Nutrient	Unit (per 100 g)	iHMNutD				Summary literature values from other reviews^1^					MILQ study^2^	
											Median (IQR)	Fold difference MILQ vs. iHMNutD^3^
		Sample size	*n* studies	Weighted mean	Pooled SD	Sample size	*n* studies	Weighted mean	Pooled SD	Fold difference select reviews vs. iHMNutD^3^		
Energy	kcal	9	208	67.44	9.16	143^4^	—	66.00	8.73	0.98	58.87 (50.44, 68.28)	0.87
Macronutrients												
Protein	g	8	199	0.93	0.14	296	4	1.10	0.17	1.18	0.82 (0.68, 1.00)	0.89
Total lipid (fat)	g	14	350	3.53	1.09	489	10	3.38	1.13	0.96	3.10 (2.33, 4.27)	0.88
Lactose	g	4	106	6.79	1.06	280	7	6.04	0.44	0.89	6.69 (6.50, 6.89)	0.99
Minerals												
Calcium	mg	5	841	31.03	5.19	1667	22	26.79	3.96	0.86	27.84 (24.15, 31.52)	0.90
Chloride	mg	5	139	40.35	11.67	108	3	38.88	9.75	0.96	—	
Copper	mg	9	907	0.044	0.020	1479	19	0.037	0.029	0.84	0.03 (0.02, 0.03)	**0.57**
Iodine	*μ*g	2	765	18.25	13.80	644	2	13.19	7.34	**0.72^^^**	Ranges from 10.96 (Denmark) to 39.09 (Bangladesh)^2^	
Iron	mg	7	231	0.050	0.038	747	12	0.058	0.055	1.15	0.02 (0.02, 0.03)	**0.48**
Magnesium	mg	5	841	3.42	0.73	1475	18	3.36	0.95	0.98	3.20 (2.72, 3.59)	0.94
Manganese	mg	1	671	0.00019	0.00017	175	5	0.52	0.24	**2736.84^^^**^5^	—	
Phosphorus	mg	2	765	14.01	2.53	972	8	13.12	2.09	0.94	14.55 (12.90, 16.39)	1.04
Potassium	mg	10	905	55.56	7.31	949	12	48.05	6.72	0.86	52.57 (47.04, 59.07)	0.95
Selenium	*μ*g	2	671	1.98	0.48	388	5	1.63	0.38	0.83	0.87 (0.68, 1.07)	**0.44**
Sodium	mg	12	924	14.73	6.99	1196	17	26.09	19.13	**1.77^^^**	10.77 (8.44, 15.13)	**0.73**
Zinc	mg	15	1071	0.19	0.09	2575	29	0.19	0.06	1.01	0.13 (0.08, 0.17)	**0.66**
Vitamins											—	
Thiamin	mg	1	13	0.026	0.004	67	1	0.024	0.004	0.92	0.01 (0.01, 0.01)	**0.41**
Riboflavin	mg	1	13	0.010	0.0001	67	1	0.033	0.004	**3.42^^^**	0.01 (0.01, 0.02)	1.12
Pantothenic Acid	mg	1	26	0.25	0.07	—	*Same study*	—	—	—	0.23 (0.17, 0.30)	0.93
Vitamin B-6	mg	1	30	0.012	0.003	71	3	0.014	0.005	1.19	0.01 (0.00, 0.01)	**0.61**
Folate	*μ*g	5	103	7.46	2.69	148	7	9.40	4.33	**1.26^^^**	—	
Water-soluble Choline	mg	2	321	11.32	3.07	301	1	11.17	3.09	0.99	—	
Total choline	mg	1	48	12.10	2.70	113	3	13.99	2.94	1.16	10.57 (8.44, 13.09)	0.87
Retinol	*μ*g	3	46	36.09	3.59	627	8	34.43	6.63	0.95	45.57 (29.17, 70.85)	**1.26**
β-carotene	*μ*g	1	13	2.84	0.11	—	*Not included*		—	—	—	
Vitamin K	*μ*g	3	79	0.19	0.15	56	2	0.23	0.2	**1.23^^^**	—	
25(OH)D	*μ*g	1	21	0.13	0.06	147^6^	7	0.024	0.03	**0.18^^^**	0.01 *(for 0‒8.5 mo, no IQR)*	**0.08**
Vitamin E	mg	1	21	0.37	0.17	766	6	0.27	0.19	**0.75^^^**	0.36 (0.17, 0.54)	0.97

Abbreviations: CI, confidence interval; HM, human milk; iHMNutD, interim human milk nutrient profile data; MILQ, mothers, infants, and lactation quality; NASEM, National Academies of Sciences, Engineering, and Medicine; 25(OH)D, 25-hydroxyvitamin D.

1 For a point of comparison, weighted mean and pooled SD values are provided where available for studies identified in other reviews converted to units per 100 g HM assuming a HM density of 1.031 g/mL. With the exception of energy and 25(OH)D, iHMNutD macronutrient, mineral, and vitamin values were compared with weighted means and pooled SDs calculated for studies identified in an evidence scan published by the NASEM in 2020 [9] (see Supplemental Table 3 for details). This evidence scan had similar inclusion criteria for HM collection and analysis as applied in iHMNutD, but it differed in other criteria, notably by including studies from other high- and middle-high income countries outside of North America, including studies of milk collected <1-mo postpartum, and excluding studies based on maternal age <18, maternal and infant general health, and multiple births. Values were not, therefore, expected to be identical but were intended as a point of comparison to help assess whether iHMNutD values were plausible.

2 For an additional point of comparison, iHMNutD values were compared with those recently published for macronutrients, and select vitamins and minerals from the MILQ study, which collected HM samples from 1242 well-nourished women from Bangladesh, Brazil, Denmark, and the Gambia over the course of the first 8.5 mo of lactation. For this comparison, the median values for 1‒6 mo were used and converted into the same units as iHMNutD as described above. MILQ differed in inclusion criteria from iHMNutD, notably by in its focus on countries outside North America, and with restrictions on characteristics such as maternal age, BMI, past or current medical problems, alcohol, smoking, diet type (excluding vegan or macrobiotic diets), micronutrient supplementation (iron and folic acid permitted in all sites and vitamin D and calcium in Bangladesh and Denmark), intake of fortified foods except iodized salt, multiple gestations, diet diversity, infant birth weight and infant congenital anomalies [23]. Values are therefore not expected to be identical but to provide a plausible range for comparison. Iodine was measured in MILQ, but reference values were not generated due to wide variability between countries; values for the range of countries are provided instead.

3 Fold difference calculated for comparative purposes as Comparator/iHMNutD. Values within 0.2-fold (20%), i.e., 0.8–1.2, were considered to be within the regular range of expected analytical variability for food composition data. Values indicated with ^ are outside this range.

4 For energy, comparison values shown are from a systematic review and meta-analysis published by Gidrewicz and Fenton in 2014 [15]. This review was limited to 24-h milk collections for energy and included studies from North America, Europe, Australia, Israel, and Japan. The review presented data from various timepoints postpartum. The 10/12-wk timepoint is shown in the table, but the 3/4-wk time point was similar: 64 (SD 9) kcal/100 g. Though not shown in the table, the review also had values for other macronutrients that were similar to iHMNutD: true protein [1.0 (SD 0.1) g/100 g], fat [3.3 (SD 0.9) g/100 g], lactose [6.5 (SD 0.7) g/100 g], calcium [25.2 (SD 5.8) mg/100 g], and phosphorus [15.5 (SD 2.9) mg/100 g] for the 10/12 wk timepoint.

5 The NASEM Evidence Scan included 1 large study from China (Qian et al. [27]) that reported a HM manganese content of 0.7‒1.9 mg/dL depending on region. This is >500x higher than the next highest value identified in the review. When this study was excluded, the NASEM weighted mean was 0.00166 (SD 0.00018) mg/100 g, which is still 8.3-fold higher than the iHMNutD value.

6 For 25(OH)D, comparison values shown are the North American values from a systematic review and meta-analysis published by Rios-Leyvraz and Yao in 2023 [16], using the 5 ng/IU conversion factor used by the authors. This review also provided values for calcium (25.32 mg /100 g; 95% CI: 24.2, 26.4 mg /100 g) and zinc (0.18 mg /100 g; 95% CI: 0.16, 0.21 mg/ 100 g) that are similar to iHMNutD.

**TABLE 3:** Comparison of interim human milk nutrient profile data values for fatty acids and cholesterol with select literature

USDA standard reference legacy		iHMNutD (g)				Percent composition^1^	Meta-analysis of global studies, (Zhang et al. [17]) (percent of fatty acids)					Large Canadian study, (Miliku et al. [18]) (percent of fatty acids)		
Nutrient	Value	Sample Size	*n* studies	Weighted mean	Pooled SD	Calculated based on iHMNutD total fat	Sample size	*n* studies	Mean (%)	SD (%)	Fold Difference Zhang vs. iHMNutD^2^	Mean (%)	SD (%)	Fold Difference Miliku vs. iHMNutD^2^
Fatty acids (g)														
SFA 4:0	0	19	1	0.00	0.00	0.00	—	—	—	—	—	—	—	—
SFA 6:0	0	48	2	0.0010	0.0010	0.03	1252	19	0.13	0.47	**4.34_^_**	—	—	—
SFA 8:0	0	48	2	0.0034	0.0015	0.10	3833	59	0.21	0.22	**2.06_^_**	—	—	—
SFA 10:0	0.063	118	4	0.034	0.010	1.03	8938	94	1.37	0.86	**1.33_^_**	0.71	0.3	**0.69^^^**
SFA 12:0	0.256	118	4	0.17	0.06	4.97	9531	108	5.7	2.81	1.15	4.8	1.57	0.96
SFA 14:0	0.321	118	4	0.20	0.07	6.09	9653	113	6.56	3.05	1.08	5.97	1.8	0.98
SFA 16:0	0.919	118	4	0.71	0.09	21.21	9838	117	21.5	4.82	1.01	20.9	2.76	0.99
SFA 18:0	0.293	118	4	0.23	0.05	6.99	9783	118	6.36	2.07	0.91	6.54	1.31	0.94
Total SFA^3^	2.01	—	—	1.35	0.14	40.43	7754	86	42.2	7.73	1.04	39.75	5	0.98
MUFA 16:1	0.129	99	3	0.09	0.02	2.61	6432	59	2.3	0.92	0.88	2.69	0.68	1.03
MUFA 18:1n‒9	1.48	118	4	1.18	0.15	35.37	6892	75	32.6	5.84	0.92	37.05	3.59	1.05
MUFA 20:1n‒9	0.04	29	1	0.012	0.003	0.36	4378	64	0.46	0.28	**1.28^^^**	—	—	—
MUFA 22:1n‒9	0	48	2	0.0030	0.0020	0.09	4984	49	0.18	0.69	**2.00^^^**	—	—	—
Total MUFA^4^	1.66	—	—	1.28	0.15	38.43	7404	86	36.3	6.46	0.94	43.06	3.59	1.12
PUFA 18:3n‒3 (ALA)	0.052	118	4	0.049	0.015	1.48	10,367	121	1.11	1.05	**0.75^^^**	1.92	0.61	**1.30^^^**
PUFA 20:3n‒3	—	58	3	0.015	0.0094	0.44	4949	51	0.1	0.12	**0.23^^^**	0.35	0.11	0.80
PUFA 22:5n‒3	0	48	2	0.0042	0.0006	0.13	—	—	—	—	—	0.13	0.05	1.03
PUFA 20:5n‒3 (EPA)	0	132	4	0.0023	0.0021	0.07	8366	97	0.12	0.67	**1.72^^^**	0.08	0.05	1.14
PUFA 22:6n‒3 (DHA)	0	145	5	0.0066	0.0049	0.20	10,083	118	0.37	0.31	**1.88^^^**	0.18	0.12	0.91
n‒3 PUFA^5^	—	—	—	0.077	0.019	2.31	5,933	68	1.88	2.63	0.81	2.39	0.7	1.04
PUFA 18:2n‒6 (LA)	0.374	118	4	0.56	0.12	16.72	10,214	117	15.7	7.15	0.94	13.62	3.01	0.81
PUFA 20:4n‒6 (ARA)	0.026	118	4	0.014	0.003	0.42	10,139	119	0.5	0.25	**1.20^^^**	0.38	0.09	0.91
PUFA 22:4n‒6	—	48	2	0.0034	0.0015	0.10	—	—	—	—	—	0.04	0.03	**0.39^^^**
n-6 PUFA^6^	—	—	—	0.58	0.12	17.24	6821	69	17.8	7.51	1.03	14.8	3.09	0.86
Total PUFA^7^	0.497	—	—	0.65	0.12	19.55	—	—	19.68	—	1.01	17.19	—	0.88
Cholesterol (mg)	14	10	1	9.83	0.37	—	1162	9	14.0	5.55	1.42	—	—	—

Abbreviations: ALA, α-linolenic acid; FAO, Food and Agriculture Organization; iHMNutD, interim human milk nutrient profile data; LA, linoleic acid.

1 For the purposes of comparison with other literature on human milk fatty acids, iHMNutD fatty acid concentrations were converted to percentage composition by dividing by the iHMNutD total fat value (3.53 g/100 g) converted to total fatty acids using a conversion factor of 0.945 as recommended by FAO for milk and milk products [14]. Values are compared for reference with global values reported in a recent systematic review and meta-analysis for mature human milk conducted by Zhang et al. [17], and with a recent large Canadian study published by Miliku et al. [18].

2 Fold difference calculated for comparative purposes as (comparator/iHMNutD). Values within 0.2-fold (20%), i.e., 0.8–1.2 were considered to be within the regular range of expected analytical variability for food composition data. Values indicated by ^ are outside this range.

3 Total SFA calculated as the sum of weighted mean values in the table for SFA 4:0, 6:0, 8:0, 10:0, 12:0, 14:0, 16:0, and 18:0.

4 Total MUFA calculated as the sum of weighted mean values in the table for MUFA 16:1, 18:1, 20:1, and 22:1.

5 Total n‒3 PUFA calculated as the sum of weighted mean values in the table for ALA, PUFA 20:3, PUFA 22:5n‒3, EPA, and DHA.

6 Total n‒6 PUFA calculated as the sum of weighted mean values in the table for LA, ARA, and PUFA 22:4n‒6.

7 Total PUFA calculated as the sum of weighted mean values in the table for all n‒6 and n‒3 PUFA.

The NASEM scanning review included comparable studies from high- and middle-high income countries on HM macronutrients, most vitamins, and minerals. The weighted means of these studies were within 20% of iHMNutD values for all of these components with the exception of iodine, manganese, sodium, riboflavin, folate, vitamin K, and vitamin E (Table 2). Energy was not captured in the NASEM scanning review, but the iHMNutD value [67.4 (SD 9.2) kcal/100 g] was similar to the concentrations reported in a 2014 systematic review and meta-analysis published by Gidrewicz and Fenton [15] [66.0 (SD 8.7) kcal/100 g], which included studies from North America, Europe, Australia, Israel, and Japan. This review also included values for lactose, protein, fat, calcium, and phosphorus, which were similar to iHMNutD values (see Table 2 footnote 4). The iHMNutD energy value was also in line with the estimated HM energy content used by the European Society for Paediatric Gastroenterology, Hepatology, and Nutrition in the proposed global standard for infant formula composition (65 kcal/100 mL) [28]. The greatest difference between the values from the NASEM scanning review and iHMNutD was for HM manganese. The iHMNutD value, based on a single study, was >2000-fold lower than the weighted mean of the studies identified in the NASEM evidence scan, whose mean was influenced by 1 study from China [27] that had a HM manganese content >500× higher than the other 4 international studies identified [[29], [30], [31], [32]]. When this study was excluded, the weighted mean of the studies in the NASEM evidence scan was still 8.3-fold higher than the iHMNutD manganese value.

The MILQ studies provided reference values for macronutrients, vitamins, and minerals based on HM samples collected in Bangladesh, Brazil, Denmark, and the Gambia. Values in iHMNutD were also within 20% of median values reported by the MILQ studies for 1‒6 mo for energy, protein, total lipid, carbohydrate, calcium, magnesium, phosphorous, potassium, riboflavin, pantothenic acid, total choline and vitamin E, but differed more substantially for copper, iron, selenium, sodium, zinc, thiamin, retinol, and 25(OH)D (Table 2). Iodine was measured in MILQ, but no reference value was generated due to wide variability between countries, although iHMNutD is within the range reported for the 4 countries.

Fatty acid and cholesterol values from iHMNutD were compared with those from a 2022 systematic review and meta-analysis of global HM lipid profiles by Zhang et al. [17]*,* and fatty acid values were also compared with those from a large recent Canadian study of HM fatty acid composition by Miliku et al. [18] to provide a North American-specific comparison (Table 3). The fatty acids making up >80% of fatty acids in HM (12:0, 14:0, 16:0, 18:0, 16:1n‒7, 18:1n‒9, linoleic acid) were <20% different between iHMNutD and the 2 comparators, and most were <10% different. Fatty acids 6:0, 8:0, 10:0, 20:1n‒9, 22:1n‒9, 20:3n‒3, arachidonic acid, EPA, and DHA were all >20% different between iHMNutD and the Zhang et al. [17] global mean, but were <20% different from Canadian values reported by Miliku et al. [18], with the exception of 10:0 (+31% difference), 22:4n‒6 (+61%), and ALA (‒30%).

## Discussion

Reliable data on HMC is needed to support many federal activities in Canada and the United States [10]. Summary statistics as nutrient concentrations were calculated for >40 nutrients, many of which have not been updated in United States or Canadian food composition databases in nearly 50 y [5,6]. Shifts in population demographics and improvements in best practices for HM collection and analysis in this period provide a strong rationale for updating reference values. iHMNutD also includes information on crucial factors needed to assess the quality and applicability of HMC data, such as the literature source, sample size, timing of milk collection, type of milk collected, analytical techniques employed, and variance, which is unavailable in the SR legacy profile. For some nutrients in the SR legacy profile, iHMNutD provides a summary of available quantitative, HM-derived human data for the first time. This includes iodine and chloride, which were not part of the SR legacy HM profile; β-carotene, vitamin E, and vitamin K, which were previously estimated based on cow’s milk; and the fatty acids EPA and DHA, which were listed at 0 g/100 g [5,6]. iHMNutD therefore includes more appropriate data for these nutrients in HM to support federal initiatives and the work of other stakeholders.

We identified no studies meeting iHMNutD inclusion criteria in the source reviews for fluoride, niacin, vitamin B-12, vitamin C, and total vitamin D, and only a single study each for manganese, β-carotene, thiamin, riboflavin, pantothenic acid, vitamin B-6, total choline, 25(OH)D, vitamin E, cholesterol, and the fatty acids 4:0 and 20:1n‒9. Although data from other countries are available for most of these nutrients from either MILQ or other studies identified in the NASEM evidence scan, which may help to fill gaps until appropriate North American data become available, there remains a lack of data for vitamin C and fluoride, and only 1 or 2 studies for several other components. Studies of HM in lactation beyond 6 mo were particularly limited, leading us not to produce values for 7‒12 mo. This is an important gap, as HMC continues to change over extended lactation, and national statistics estimate that >30% of Canadian and United States infants consume human milk up to ≥12 mo of age [[33], [34], [35]].

Although for most nutrients, iHMNutD aligned broadly with comparable literature, some discrepancies were observed. Using a >20% difference as the threshold for meaningful variation (based on typical variability in food composition data), iHMNutD differed from the weighted mean of studies in the NASEM evidence scan for iodine, manganese, sodium, riboflavin, folate, vitamin K, and vitamin E; from the MILQ study for copper, iron, selenium, sodium, zinc, thiamin, retinol, and 25(OH)D; and from both the Zhang et al. [17] global analysis of HM fatty acids and the large Canadian comparison paper for fatty acids 10:0, 22:4n‒6, and ALA.

The NASEM evidence scan, the MILQ study, and the Zhang et al. [17] meta-analysis all included participants from countries outside North America, whereas iHMNutD focused on North American populations. The MILQ study also applied additional inclusion criteria that restricted participant characteristics such as BMI, maternal medical conditions, and supplement use. Diet, ethnicity, BMI, maternal chronic disease, and supplement use have all been shown to be associated with HMC [4,11], but these factors were not restricted in iHMNutD because they occur in the general North American population and therefore reflect current exposures, which was the intent of these values. These factors may partly explain differences between iHMNutD and the comparison studies, particularly for components such as iodine, B vitamins, selenium, and fatty acids, which are strongly influenced by diet [4]. This was especially evident for fatty acids, for which many differences were observed between iHMNutD and global means, but very few when compared with the Canadian study [17,18]. Remaining differences in fatty acids relative to the Canadian study may also be partly explained by dietary patterns. For example, iHMNutD values for ALA were derived largely from United States studies, whereas Canada has higher ALA intake due to the widespread use of canola oil [36].

Another possible explanation for differences between iHMNutD and other data sources is the timing of milk collection. The MILQ study collected repeated samples ≤8.5 mo of lactation, and observed gradual decreases in the HM content of several nutrients [19]. iHMNutD included a heavily-weighted study from earlier in lactation for copper, zinc, and sodium, which may partly explain its higher concentrations compared to the MILQ 1‒6 mo medians.

For some nutrients, comparing iHMNutD with the NASEM evidence scan and MILQ helps to contextualize discrepancies. For example, although the iHMNutD vitamin E concentration (0.37 mg/100 g, based on 1 study) was higher than the NASEM weighted mean (0.27 mg/100 g, based on 6 studies), it was very similar to the 1‒6 mo median reported in MILQ (0.36 mg/100 g), which may increase confidence in this value. Similarly, the iHMNutD riboflavin value, based on a single study of 13 participants [37], was >3-fold lower than the single Serbian study identified in the NASEM evidence scan [38], but was close to the reference value reported by MILQ (0.010 compared with 0.011 mg/100 g) [20]. In contrast, the iHMNutD thiamin value (0.026 mg/ 100 g), although close to the mean of the single other study identified in the NASEM evidence scan (0.024 mg/100 g [38]), was substantially higher than the value reported in MILQ (0.01 mg/100 g) [21]. For 25(OH)D, the same article was included in both iHMNutD and the NASEM evidence scan [39]; however, its reported value [0.13 (SD 0.06) g/100 g] was much higher than the value for North American studies from a 2023 systematic review and meta-analysis by Rios-Leyvraz and Yao [16] [0.024 (SD 0.03) μg/100 g], which did not restrict studies to full breast expression, and higher than the value reported in MILQ (0.01 μg/100 g). This suggests that the iHMNutD value may not be representative. The iHMNutD retinol value [36.1 (SD 3.6) μg/100g], was similar to the weighted mean of studies identified in the NASEM evidence scan [34.4 (SD 6.6) μg/100 g based on 8 studies], and to the mean for early mature milk reported in a recent global systematic review and meta-analysis that did not restrict to full breast expression (39.0; 95% confidence interval: 33.2, 44.8) [40], but was lower than the reference value reported by MILQ (45.6; IQR: 29.2‒70.9, μg/100 g). These discrepancies, together with the limited available literature for iHMNutD, introduce uncertainty.

Some nutrients showing discrepancies lacked comparison data from MILQ. The iHMNutD cholesterol value [9.8 (SD 0.4) g/100 g], based on only a single study of 10 participants, was >20% lower than the existing SR legacy value (14.0 g/100 g), and was also lower than the global mean reported by Zhang et al. [17] [14.0 (SD 5.6) g/100 g]. This difference, together with the very limited data supporting iHMNutD, raises concerns about its suitability for updating food composition databases. The iHMNutD manganese value was also based on a single study, although this study included a large sample size (*n* = 671) and used inductively coupled plasma-mass spectrometry, which is considered optimal for trace element analysis [11]. This iHMNutD value was >8-fold lower than the weighted mean of studies identified in the NASEM evidence scan, even after excluding a single potential outlier study; however, those comparison studies were themselves >10-fold lower than the SR legacy value. This suggests that the SR legacy value for HM manganese is an overestimate, but the wide variability in the literature makes it uncertain whether the lower iHMNutD value is an appropriate replacement.

Flags for values based on a single study, a small sample size, and/or data from before the year 2000 were proposed so that data users would be aware of these limitations. Further consultations are underway with federal stakeholders to determine the suitability of iHMNutD values, in whole or in part, to update the outdated FoodData Central and Canadian Nutrient File values, especially where there is uncertainty. In cases of greater uncertainty, food composition databases may benefit from incorporating values from other international studies or from presenting international values alongside domestic data for comparison.

Although iHMNutD offers significant improvements over the data that was used to generate the existing SR legacy values [7], it also has several limitations. The iHMNutD values were generated based on 3 recent reviews [[7], [8], [9]] rather than a new systematic review and meta-analysis conducted for this purpose. These reviews differed in search strategy and inclusion criteria, which raises the possibility that some relevant references may have been excluded, either in the initial searches or in our subsequent harmonization of the inclusion criteria. Although we attempted to mitigate this limitation by choosing reviews that overlapped in time frame and by employing ≥2 independent reviewers for study selection and data extraction, a future systematic review conducted entirely for the purposes of iHMNutD would be beneficial. Our process also did not capture most studies published after the time frame covered by the source systematic reviews (1980‒2022). In addition, although iHMNutD provides new values for many nutrients, some nutrients still remain without data. Future discussions with stakeholders will determine whether to retain the SR legacy value in these cases, or to use data from other countries.

In conclusion, iHMNutD offers critical updates to the HMC profile through a transparent, data-backed foundation. These efforts can support better-informed nutritional policies in North America until more comprehensive studies are available in the future.

## Author contributions

The authors’ responsibilities were as follows – All coauthors designed this research; ST, KEH: conducted the research and analyzed data with oversight of AJV, KC, KG, KAZ, TI, MS; All coauthors contributed to the manuscript writing; and all authors: read and approved the final manuscript.

## Data availability

Data described in the manuscript, code book, and analytic code will be made available upon request.

## Funding

The authors reported no funding received for this study.

## Conflict of interest

The authors report no conflicts of interest.
