# Supplementary material for: Literature-based human milk nutrient composition values for use in North American food composition databases
Source: Am J Clin Nutr. 2026 Apr 7;123(5):101252. doi: 10.1016/j.ajcnut.2026.101252 (PMC13197950; doi:10.1016/j.ajcnut.2026.101252)
Supplement: Multimedia component 1 [file mmc1.pdf]

Supplemental Material

Table of Contents

Supplemental Table 1 – list of all nutrients included and preferred analytical methods[1]. 2
Supplemental Table 2 – Studies included in iHMNut for the 0-6 months range 5
A: Studies included in iHMNut for the 0-6 Months for Energy and Macronutrients 5
B: Studies included in iHMNut 0-6 Months for Trace Elements 9
C: Studies included in iHMNut 0-6 Months for Macroelements 12
D: Studies included in iHMNut 0-6 Months for Water Soluble Vitamin 16
E: Studies included in iHMNut 0-6 Months for Fat Soluble Vitamins 17
F: Studies included in iHMNut Fatty Acids and Cholesterol 18
Supplemental Table 3: Studies from NASEM scanning review used in the comparison weighted means and pooled standard deviations cited in Table 2[1] 20

Supplemental Table 1 – list of all nutrients included and preferred analytical methods[1].

Inclusion Criteria for Sample Type and Analytical Techniques[1]		
**Component**	**Preferred Sample Type**	**Preferred Analytical technique**
Energy	Full breast expression, 24-hour collection, or weighted combined aliquots of fore-and hindmilk from one breast at each pumping over 24 hours; standardized by time of day, collection mode, collection breast, and time since last feed/expression	Calorimetry, calculation from proximates
Protein	Any milk type standardized by time of day, collection mode, collection breast, and time since last feed/expression	Kjeldahl with correction for nonprotein nitrogen by acid precipitation
Total lipid (fat)	Full breast expression, 24-hour collection, or weighted combined aliquots of fore-and hindmilk from one breast at each pumping over 24 hours; standardized by time of day, collection mode, collection breast, and time since last feed/expression	Solvent extraction (e.g Folch, Bligh-Dyer, Rose-Gottlieb), followed by weighing, or quantitative GC-FID, GC-MS or similar
Fatty acid panel	Full breast expression, 24-hour collection, or weighted combined aliquots of fore-and hindmilk from one breast at each pumping over 24 hours; standardized by time of day, collection mode, collection breast, and time since last feed/expression	Quantitative (units in mg, ug, ng/mL or per g), or weight percent with quantity of total fat reported, GC-FID, GC-MS or similar
Cholesterol	Full breast expression, 24-hour collection, or weighted combined aliquots of fore-and hindmilk from one breast at each pumping over 24 hours; standardized by time of day, collection mode, collection breast, and time since last feed/expression	Saponification + GC-FID, GC-MS or similar
Carbohydrate (lactose)	Any milk type standardized by time of day, collection mode, collection breast, and time since last feed/expression	LC-MS, HPLC (with FLD, MS detection or similar)
Lactose	Any milk type standardized by time of day, collection mode, collection breast, and time since last feed/expression	LC-MS, HPLC (with FLD, MS detection or similar)
**Minerals**		
Calcium, Ca	Any milk type, use trace-element free supplies	ICP-MS
Iron, Fe	Any milk type, use trace-element free supplies	ICP-MS, AAS (atomic absorption spectrometry), inductively coupled argon plasma
Magnesium, Mg	Any milk type, use trace-element free supplies	ICP-MS
Phosphorus, P	Any milk type, use trace-element free supplies	ICP-MS
Potassium, K	Any milk type, use trace-element free supplies	ICP-MS, AAS, flame photometry
Sodium, Na	Any milk type, use trace-element free supplies	ICP-MS, flame atomic emission spectrometry, AAS
Zinc, Zn	Any milk type, use trace-element free supplies	AAS, ICP-AES, ICP-MS
Copper, Cu	Any milk type, use trace-element free supplies	AAS, ICP-AES, ICP-MS
Manganese, Mn	Any milk type, use trace-element free supplies	ICP-MS
Selenium, Se	Any milk type, use trace-element free supplies	AAS, ICP-MS
Iodine	Any milk type, use trace-element free supplies	ICP-MS, ICP-AES
Chloride	Any milk type, use trace-element free supplies	Chloridometer, colorimetric titration by chloride counter, potentiometric method
Fluoride	Any milk type, use trace-element free supplies	Fluoride-specific electrode
Vitamin C	Combined morning and evening sample, or afternoon and evening samples preferred.	HPLC (with FLD, UV detection or similar)
Thiamin	Combined morning and evening sample, or afternoon and evening samples preferred.	HPLC-FLD, thiochrome method, other chromatographic separation with fluorescence detection, *L. viridescens* microbial assay
Riboflavin	Combined morning and evening sample, or afternoon and evening samples preferred.	HPLC (with FLD, UV detection or similar), UPLC-MS/MS
Niacin (various forms)	Combined morning and evening sample, or afternoon and evening samples preferred.	*L. arabinosus* microbial assay, LC-MS/MS, UPLC-MS/MS
Pantothenic acid	Combined morning and evening sample, or afternoon and evening samples preferred.	Microbiological assays, chromatographic separation (GC, LC, UPLC) followed by UV or MS/MS detection
Vitamin B-6	Combined morning and evening sample, or afternoon and evening samples preferred.	HPLC, LC-based method of *saccharomyces uvarum,* UPLC-MS/MS
Biotin	Combined morning and evening sample, or afternoon and evening samples preferred.	Microbiological assays (*L. arabinosus and L. plantarum),* LC-MS/MS, ULC-MS/MS

Folate	Combined morning and evening sample, or afternoon and evening samples preferred.	Microbiological assay with *L. casei* or similar, LC-MS/MS
Vitamin B-12	Combined morning and evening sample, or afternoon and evening samples preferred.	*L. leichmanii* microbial assay, competitive chemiluminescence enzyme immunoassay, competitive protein binding assay
Retinol	Full breast expression, 24-hour collection, or weighted combined aliquots of fore-and hindmilk from one breast at each pumping over 24 hours; standardized by time of day, collection mode, collection breast, and time since last feed/expression	HPLC (with FLD, UV detection or similar), LC-MS/MS
Carotene, beta	Full breast expression, 24-hour collection, or weighted combined aliquots of fore-and hindmilk from one breast at each pumping over 24 hours; standardized by time of day, collection mode, collection breast, and time since last feed/expression	HPLC (with FLD, UV detection or similar), LC-MS/MS
Carotene, alpha	Full breast expression, 24-hour collection, or weighted combined aliquots of fore-and hindmilk from one breast at each pumping over 24 hours; standardized by time of day, collection mode, collection breast, and time since last feed/expression	HPLC (with FLD, UV detection or similar), LC-MS/MS
Cryptoxanthin, beta	Full breast expression, 24-hour collection, or weighted combined aliquots of fore-and hindmilk from one breast at each pumping over 24 hours; standardized by time of day, collection mode, collection breast, and time since last feed/expression	HPLC (with FLD, UV detection or similar), LC-MS/MS
Lycopene	Full breast expression, 24-hour collection, or weighted combined aliquots of fore-and hindmilk from one breast at each pumping over 24 hours; standardized by time of day, collection mode, collection breast, and time since last feed/expression	HPLC (with FLD, UV detection or similar), LC-MS/MS
Lutein	Full breast expression, 24-hour collection, or weighted combined aliquots of fore-and hindmilk from one breast at each pumping over 24 hours; standardized by time of day, collection mode, collection breast, and time since last feed/expression	HPLC (with FLD, UV detection or similar), LC-MS/MS
Zeaxanthin	Full breast expression, 24-hour collection, or weighted combined aliquots of fore-and hindmilk from one breast at each pumping over 24 hours; standardized by time of day, collection mode, collection breast, and time since last feed/expression	HPLC (with FLD, UV detection or similar), LC-MS/MS
Vitamin K	Full breast expression, 24-hour collection, or weighted combined aliquots of fore-and hindmilk from one breast at each pumping over 24 hours; standardized by time of day, collection mode, collection breast, and time since last feed/expression	HPLC-FLD, LC-MS/MS
Vitamin E	Full breast expression, 24-hour collection, or weighted combined aliquots of fore-and hindmilk from one breast at each pumping over 24 hours; standardized by time of day, collection mode, collection breast, and time since last feed/expression	HPLC (with FLD, UV detection or similar), LC-MS/MS
Vitamin D2	Full breast expression, 24-hour collection, or weighted combined aliquots of fore-and hindmilk from one breast at each pumping over 24 hours; standardized by time of day, collection mode, collection breast, and time since last feed/expression	LC/MS/MS, UPLC-MS/MS, HPLC, CPBA (competitive protein binding assay)
Vitamin D3	Full breast expression, 24-hour collection, or weighted combined aliquots of fore-and hindmilk from one breast at each pumping over 24 hours; standardized by time of day, collection mode, collection breast, and time since last feed/expression	LC/MS/MS, UPLC-MS/MS, HPLC, CPBA (competitive protein binding assay)
25-OH-D2	Full breast expression, 24-hour collection, or weighted combined aliquots of fore-and hindmilk from one breast at each pumping over 24 hours; standardized by time of day, collection mode, collection breast, and time since last feed/expression	LC/MS/MS, UPLC-MS/MS, HPLC, CPBA (competitive protein binding assay)
25-OH-D3	Full breast expression, 24-hour collection, or weighted combined aliquots of fore-and hindmilk from one breast at each pumping over 24 hours; standardized by time of day, collection mode, collection breast, and time since last feed/expression	LC/MS/MS, UPLC-MS/MS, HPLC, CPBA (competitive protein binding assay)
Choline, total	Combined morning and evening sample, or afternoon and evening samples preferred.	LC-MS/MS, GC-MS, radioimmunoassay, UPLC-MS/MS
Carnitine	Combined morning and evening sample, or afternoon and evening samples preferred.	Chromatographic separation followed by MS, UPLC-MS/MS
Ash	Full breast expression, 24-hour collection, or weighted combined aliquots of fore-and hindmilk from one breast at each pumping over 24 hours; standardized by time of day, collection mode, collection breast, and time since last feed/expression	Ash determination (e.g incineration and weighing)

Moisture	Full breast expression, 24-hour collection, or weighted combined aliquots of fore-and hindmilk from one breast at each pumping over 24 hours; standardized by time of day, collection mode, collection breast, and time since last feed/expression	Loss on drying

1Adapted from Mohr *et al.* and the NASEM Scanning review (1, 2). Chromatographic separation can include techniques such as gas chromatography (GC), liquid chromatography (LC), high-performance liquid chromatography (HPLC), ultrahigh-performance liquid chromatography (UHPLC), etc.

GC-FID: Gas Chromatography-Flame Ionization Detector; GC-MS: Gas Chromatography-Mass Spectrometry; LC-MS: Liquid Chromatography-Mass Spectrometry; HPLC: High-Performance Liquid Chromatography; FLD: Fluorescence Detector; MS: Mass Spectrometry; ICP-MS: Inductively Coupled Plasma-Mass Spectrometry; AAS: Atomic Absorption Spectrometry; ICP-AES: Inductively Coupled Plasma-Atomic Emission Spectrometry; UPLC-MS/MS: Ultra-Performance Liquid Chromatography-Tandem Mass Spectrometry; UV: Ultraviolet; CPBA: Competitive Protein Binding Assay

Supplemental Table 2 – Studies included in iHMNut for the 0-6 months range

A: Studies included in iHMNut for the 0-6 Months for Energy and Macronutrients

Nutrient	Unit per 100g	Reference	Sample Size	Mean	SD	Analytical Method	Location	Time post-partum	Milk Type	Time of Day
**Energy**										
Energy	kcal	Nommsen et al., 1991 (3)	58	67.60	6.50	Calculated from proximates	USA	3 Months	Full breast expression	Each feeding over a 24-hour period
		Butte et al., 1990 (4)	20	64.00	7.07	Bomb calorimeter	USA	30 – 120 days	The infant nursed from one breast and milk was expressed from the other	Each feeding every 24 hours
		Gross et al., 1980 (5)	18	67.07	11.74	Calculated from proximates	USA	21-28 days	Full breast expression	Morning
		Butte et al., 1984 (6)	13	65.62	9.89	Bomb calorimeter	USA	28-84 days	Full breast expression	Between 8 am – 12 PM
		Ferris et al., 1988 (7)	12	75.43	10.71	Calculated from proximates	USA	42-112 days	Full breast expression	1.5 hours after a morning (9:30 – 10:30 AM) and an afternoon (1:30-2:30 PM) nursing
		Garza and Butte et al., 1986 (8)	20	70.47	9.35	Bomb calorimeter	USA	30-120 days	The infant was offered one breast and milk was expressed from the other breast	Every 24-hour period
		Dewey and Lonnerdal et al., 1983 (9)	20	73.59	14.04	Calculated from proximates	USA	30-180 days	Full breast expression	Second feeding of the morning
		Butte et al., 1984 (10)	41	64.50	8.79	Bomb calorimeter	USA	30-120 days	The infant was offered one breast, and the other breast was emptied of its entire contents	Each feeding over a 24-hour period

		Garza et al., 1983 (11)	6	55.87	3.98	Bomb calorimeter	USA	180 days	Full breast expression	Between 8 am – 12 PM
True Protein	**Macronutrients** g									
		Butte et al., 1990 (4)	20	0.92	0.21	Kjeldahl with correction for non-protein nitrogen	USA	30-120 days	The infant nursed from one breast and milk was expressed from the other breast with an electric breast pump	Each feeding over 24 hours
		Stuff and Nichols et al., 1989 (12)	45	0.79	0.13	Kjeldahl with correction for non-protein nitrogen	USA	112-252 days	24-hour milk sample	Not available
		Gross et al., 1980 (5)	18	1.40	0.18	Kjeldahl with correction for non-protein nitrogen	USA	21-28 days	Full breast expression	morning
		Butte et al., 1984 (6)	13	0.95	0.15	Kjeldahl with correction for non-protein nitrogen	USA	28-84 days	Full breast expression	Between 8 am – 12 PM
		Butte et al., 1984 (10)	41	0.89	0.12	Kjeldahl with correction for non-protein nitrogen	USA	30-120 days	Full breast expression	Each feeding over a 24-hour period
		Garza et al., 1983 (11)	6	0.82	0.04	Kjeldahl with correction for non-protein nitrogen	USA	180 days	Full breast expression	Between 8 am – 12 PM
		Dewey et al., 1994 (13)	33	0.88	0.10	Kjeldahl with correction for non-protein nitrogen	USA	6-8 weeks	Full breast expression	Each feeding over a 24-hour period
		McCrory et al., 1999 (14)	23	0.88	0.13	Kjeldahl with correction for non-protein nitrogen	USA	8-16 weeks	Full breast expression	Each feeding over a 24-hour period

Total Fat	g	Clark et al., 1982 (15)	10	4.49	0.53	Folch	USA	42-112 days	Full breast expression	9:30 – 11:30 AM and 1:30 – 3:30 PM
		Nommsen et al., 1991(3)	58	3.51	0.67	Folch	USA	3 Months	Full breast expression	Each feeding over a 24-hour period
		Butte et al., 1990 (4)	20	3.08	0.81	Roese-Gottlieb	USA	30-120 days	The infant nursed from one breast and milk was expressed from the other breast with an electric pump	Each feeding over a 24-hour period
		Butte et al., 1984 (6)	13	4.26	1.14	Roese-Gottlieb	USA	28-84 days	Full breast expression	Between 8 am – 12 PM
		Stuff and Nichols et al., 1989 (12)	45	3.04	0.83	Roese-Gottlieb	USA	112-252 days	24-hour milk sample	At each feeding over a 24-hour period
		Gross et al., 1980 (5)	18	3.83	1.37	Roese-Gottlieb	USA	21-28 days	Full breast expression	morning
		Ferris et al., 1988 (7)	12	4.78	1.11	Folch	USA	42-112 days	Full breast expression	90 minutes after a morning (9:30-10:30 AM) and afternoon (1:30 – 2:30 PM nursing)
		Butte et al., 1984 (10)	41	3.44	0.84	Roese-Gottlieb	USA	30-120 days	Full breast expression	At each feeding over a 24-hour period
		Garza et al., 1983 (11)	6	3.49	0.78	Roese-Gottlieb	USA	180 days	Full breast expression	8 AM – 12 PM
		Clark et al., 1983 (16)	10	4.49	0.55	Folch	USA	42-112 days	Full breast expression	Morning nursing (9:30- 11:30 AM) and afternoon nursing (1:30-3:30 PM)
		Glew et al., 2008 (17)	29	3.27	2.27	Folch	USA	30-180 days	10-15 ml were collected using a manual pump	7 AM – 10:30 PM
		Glew et al., 2011 (18)	19	4.53	1.84	Folch	USA	30-180 days	Infant was offered one breast, and the contents of the	8:30 – 10:30 AM

									other breast were expressed with an electrical pump	
		Dewey et al., 1994 (13)	33	3.17	0.49	Folch	USA	6-8 weeks	Full breast expression	24h
		McCrory et al., 1999 (14)	23	3.31	0.58	Folch	USA	8-16 weeks	Full breast expression	Each feeding over a 24-hour period
Lactose	g									
		Goran et al., 2017 (19)	25	7.57	0.78	LC-MS	USA	1 Month	Full breast expression	8-10 AM
		Berger et al., 2018 (20)	41	6.62	1.55	LC-MS	USA	6 weeks	Full breast Expression	6 AM
		Nijman et al., 2018 (21)	10	5.50	0.09	HPAEC-PAD	USA	42 days	Milk collection from one breast using a manual breast pump	Morning
		Wack et al., 1997 (22)	30	6.79	0.34	HPLC	USA	61-120 and 121-180 d time points used	Full breast expression	10:00-14:00

B: Studies included in iHMNut 0-6 Months for Trace Elements

Nutrient	Unit per 100 g	Reference	Sample Size	Mean	SD	Analytical Method	Location	Time post-partum	Milk Type	Time of day
Iron	mg	Feeley et al., 1983 (23)	105	0.08	0.05	ICP-MS	USA	30-45 day	1/3 fore milk, 1/3 halfway through feeding, 1/3 hind milk	Late evening and early morning
		Dewey et al., 1984 (24)	12	0.02	0.01	AAS and AES	USA	120 – 330 days	Full breast expression	Second feeding of the morning
		Dewey and Lonnerdal et al., 1983 (9)	20	0.02	0.01	AAS	USA	30 – 180 days	Full breast expression	Second feeding of the morning
		Garza et al., 1983 (11)	6	0.003	0.001	AAS	USA	180 days	Full breast expression	8am – 12 PM
		Hannan et al., 2009 (25)	17	0.04	0.06	AAS	USA	30-90 days	20-30 ml of milk was collected by manual expression	Not reported
		Picciano et al., 1981 (26)	26	0.04	0.01	AAS	USA	1-3 months	Foremilk	Morning, midday, and evening (over 3 days)
		Butte et al., (1987) (27)	45	0.02	0.01	AAS	USA	1-4 months	Full breast expression	Each feed over 24 hours
Zinc	mg	Friel et al., 1999 (28)	17	0.23	0.11	ICP-MS	Canada	7-84 days	About 15 ml of milk from one whole breast expression	10 AM – 2 PM
		Feeley et al., 1983 (23)	105	0.29	0.12	ICP-MS	USA	30-45 days	1/3 fore milk, 1/3 halfway through feeding, 1/3 hind milk	Late evening and early morning
		Hopperton et al. 2024 (29)	672	0.17	0.08	ICP-MS	Canada	34 days	Hand expression of fore and hind milk over multiple days	Not reported
		Dewey et al., 1984 (24)	12	0.09	0.05	AAS and AES	USA	120 – 330 days	Full breast expression	Second feeding of the morning

		Butte et al., 1984 (6)	13	0.20	0.08	AAS and colorimetry	USA	28 – 84 days	Full breast expression	8 AM – 12 PM
		Dewey and Lonnerdal, 1983 (9)	20	0.13	0.04	AAS	USA	30- 180 days	Full breast expression	Second feeding of the morning
		Garza et al., 1983 (11)	6	0.12	0.02	AAS	USA	180 days	Full breast expression	8 AM – 12 PM
		Casey et al., 1989 (30)	13	0.20	0.08	AAS	USA	4- 360 days	Manual expression from both breasts	Midmorning feed
		Hannan et al., 2009 (25)	17	0.20	0.01	AAS	USA	30-90 days	20-30 ml of milk was collected by manual expression	Not reported
		Casey et al., 1985 (31)	9	0.29	0.32	AAS	USA	28 days	Manual expression from both breasts	Mid-morning
		Picciano et al., 1981 (26)	26	0.20	0.11	AAS	USA	1-3 months	Foremilk	Morning, midday, and evening (over 3 days)
		Hunt et al., (2005) (32)	45	0.16	0.03	ICAP-ES	USA	1-4 months	Full breast expression	All feeds over 24 hours
		Butte et al., (1987) (27)	45	0.15	0.06	AAS	USA	1-4 months	Full breast expression	Each feed over 24 hours
		Krebs et al., 1985 (33)	32	0.23	0.11	AAS	USA	1st and 2nd months	Foremilk, mid-feed, hindmilk	
		Krebs et al., 1985 (34)	39	0.26	0.08	AAS	USA	1 month	Manual expression, type not specified	Multiple collections over a month averaged together
Copper	mg	Friel et al., 1999 (28)	17	0.04	0.03	ICP-MS	Canada	7-84 days	About 15 ml of milk from one whole breast collection was taken	10 am – 2 pm
		Feeley et al., 1983 (23)	105	0.08	0.05	ICP-MS	USA	30-45 days	1/3 fore milk, 1/3 milk halfway through feeding, 1/3 hind milk	Late evening feeding and early morning feeding
		Hopperton et al. 2024 (29)	660	0.04	0.01	ICP-MS	Canada	34 days	Hand expression of hind and fore milk over multiple days	Not reported

		Dewey et al., 1984 (24)	12	0.02	0.01	AAS and AES	USA	120-330 days	Full breast expression	Second feeding of the morning
		Dewey and Lonnerdal., 1983 (9)	20	0.03	0.01	AAS	USA	30-180 days	Full expression (manual) from one breast	Second feeding of the morning
		Casey et al., 1989 (30)	13	0.03	0.01	AAS	USA	4-360 days	Except for the first 1-2 days when a breast pump was used, milk samples (2-5 ml) were obtained by manual expression into prewashed plastic vials	Mid-morning feed
		Casey et al., 1985 (31)	9	0.04	0.02	FES-AES	USA	28 days	Mothers hand expressed milk (5 ml)	Mid-morning feed
		Picciano et al., 1981 (26)	26	0.02	0.01	AAS	USA	1-3 months	Foremilk	Morning, midday, and evening (over 3 days)
		Butte et al., (1987) (27)	45	0.03	0.01	AAS	USA	1-4 months	Full breast expression	Each feed over 24 hours
Selenium	mcg	Hannan et al., 2009 (25)	17	1.53	0.31	AAS	USA	30-90 days	20-30 ml of milk was collected either by manual expression into pre-washed plastic vials or via a pre-washed trace mineral free breast pump and container	Not reported
		Hopperton et al. 2024 (29)	654	1.99	0.48	ICP-MS	Canada	34 days	Hand expression of hind and fore milk over multiple days	Not reported
Manganese	mg	Hopperton et al. 2024 (29)	671	0.0002	0.0002	ICP-MS	Canada	34 days	Hand expression of hind and fore	Not reported

									milk over multiple days	
Iodine	mcg	Hopperton et al., 2024 (29)	660	18.00	14.81	ICP-MS	Canada	34 days	Hand expression of hind and fore milk over multiple days	Not reported
		Bertinato et al., 2022 (35)	105	19.80	11.11	ICP-MS	Canada	1 Month	Full breast expression	Not reported

C: Studies included in iHMNut 0-6 Months for Macroelements

Nutrient	Unit per 100g	Reference	Sample Size	Mean	SD	Analytical Method	Location	Time post-partum	Milk Type	Time of day
Calcium	mg	Friel et al., 1999 (28)	17	27.96	3.69	ICP-MS	Canada	7-84 days	15 ml from one whole breast expression	10 am – 2pm
		Feeley et al., 1983 (36)	105	26.20	6.14	ICP-MS	USA	30-45 days	Equal volumes of milk from the beginning, middle, and end of a feeding	Morning and evening feeding
		Hopperton et al., 2024 (29)	660	32.19	5.27	ICP-MS	Canada	34 days	Hand expression of hind and fore milk over multiple days	Not reported
		Hunt et al., (2005) (32)	45	26.62	1.12	ICAP-ES	USA	1-4 months	Full breast expression	All feeds over 24 hours
		Fly et al., (1998) (37)	14	30.20	1.17	ICP-AES	USA	2-8 months	Foremilk from both breasts	Not reported
Magnesium	mg	Friel et al., 1999 (28)	17	2.91	0.51	ICP-MS	Canada	7-84 days	About 15 ml of milk from one whole breast expression	10 AM – 2 PM

		Feeley et al., 1983 (36)	105	5.00	1.26	ICP-MS	USA	30-45 days	Equal volume of milk from the beginning, middle, and end of a feeding by either a hand breast pump or by manual expression	One evening feeding and one morning feeding
		Hunt et al., (2005) (32)	45	2.99	0.21	ICAP-ES	USA	1-4 months	Full breast expression	All feeds over 24 hours
		Hopperton et al., 2024 (29)	660	3.20	0.64	ICP-MS	Canada	34 days	Hand expression of hind and fore milk over multiple days	Not reported
		Fly et al., (1998) (37)	14	3.58	0.17	ICP-AES	USA	2-8 months	Foremilk from both breasts	Not reported
Potassium	mg	Wack et al., 1997 (22)	30	50.44	9.19	ICP-MS	USA	61-120 and 121-180 d time points used	Full breast expression	Between 10:00 AM – 2 PM
		Hopperton et al., 2024 (29)	660	58.41	7.61	ICP-MS	Canada	34 days	Hand expression of hind and fore milk over multiple days	Not reported
		Dewey et al., 1984 (24)	12	39.77	7.10	AAS and AES	USA	120 – 330 days	Full breast expression	Second feeding of the morning
		Gross et al., 1980 (5)	18	58.25	8.54	AAS and colorimetry	USA	21-28 days	Full breast expression	morning
		Dewey and Lonnerdal, 1983 (9)	20	45.72	7.60	AAS	USA	30-180 days	Full expression from one breast	Second feeding of the morning
		Picciano et al., 1981 (26)	26	42.03	8.42	AAS	USA	1-3 months	Foremilk	Morning, midday, and evening (over 3 days)
		Fly et al., (1998) (37)	14	44.71	2.35	ICP-AES	US	2-8 months	Foremilk from both breasts	Not reported
		Morriss et al., (1986) (38)	52	48.54	1.90	Flame photometry	USA	120-180 days	Full breast expression	Morning
		Butte et al., (1987) (27)	45	44.25	5.53	AAS	USA	1-4 months	Full breast expression	Each feed over 24 hours
		Keenan et al., 1982 (39)	28	54.99	5.88	Flame photometry	USA	3.5-18 weeks	Foremilk	Every 4h for 24 hours

Sodium	mg	Wack et al., 1997 (22)	30	14.45	7.17	ICP-MS	USA	61-120 and 121-180 d time points used	Full breast expression	Between 10:00 AM – 2PM
		Hopperton et al., 2024 (29)	660	15.27	7.12	ICP-MS	Canada	34 days	Hand expression of hind and fore milk over multiple days	Not reported
		Dewey et al., 1984 (24)	12	9.46	5.14	AAS and AED	USA	120-330	Full expression from one breast	Second feeding of the morning
		Gross et al., 1980 (5)	18	21.53	13.16	AAS and colorimetry	USA	21-28 days	Full expression of both breasts by manual or mechanical expression	Morning
		Butte et al., 1984 (6)	13	15.49	5.11	AAS and colorimetry	USA	21-84 days	Full expression of one breast	At least two hours after a feeding between 8 am and noon
		Dewey and Lonnerdal, 1983 (9)	20	18.59	14.50	AAS	USA	30-180 days	Full expression (manual) from one breast	Second feeding of the morning
		Garza et al., 1983 (11)	6	13.19	1.55	AAS	USA	180 days	Full breast expression	3-4 hours after previous nursing between the hours of 8 am and noon
		Morriss et al., 1986 (38)	52	10.70	2.23	Flame photometry	USA	120-180 days	Full breast expression	Morning
		Picciano et al., 1981 (26)	26	12.87	4.91	AAS	USA	1-3 months	Foremilk	Morning, midday, and evening (over 3 days)
		Fly et al., (1998) (37)	14	11.15	1.07	ICP-AES	US	2-8 months	Foremilk from both breasts	Not reported
		Butte et al., (1987) (27)	45	11.20	3.00	AAS	USA	1-4 months	Full breast expression	Each feed over 24 hours
		Keenan et al., 1982 (39)	28	14.05	5.57	Flame photometry	USA	3.5-18 weeks	Foremilk	Every 4h for 24 hours
Phosphorus	mg	Feeley et al., 1983 (36)	105	13.30	3.76	ICP-MS	USA	30-45 days	Equal volumes of milk from the beginning, middle, and end of a feeding	One morning feeding and one evening feeding

		Hopperton et al. , 2024 (29)	660	14.12	2.28	ICP-MS	Canada	34 days	Hand expression of hind and fore milk over multiple days	Not reported
Chloride	mg	Wack et al., 1997 (22)	30	38.80	12.61	Potentiometric method	USA	61-120 and 121-180 d time points used	Full expression from one breast	10 am – 2pm
		Allen et al., 1991 (40)	13	48.15	7.35	Ion selective electrode and colorimetry	USA	21-180 days	Full breast expression	Morning feed
		Gross et al., 1980 (5)	18	48.72	25.16	AAS and colorimetry	USA	21-28 days	Full breast expression	Morning
		Morriss et al., (1986) (38)	52	36.10	3.44	Colorimetric amperometric titration	USA	120-180 days	Full breast expression	Morning
		Picciano et al., 1981 (26)	26	40.92	8.10	Ion electrode	USA	1-3 months	Foremilk	Morning, midday, and evening (over 3 days)

D: Studies included in iHMNut 0-6 Months for Water Soluble Vitamin

Nutrient	Unit per 100 g	Reference	Sample Size	Mean	SD	Analytical Method	Location	Time post-partum	Milk Type	Time of Day
Thiamin	mg	Lima et al., 2020 (41)	13	0.026	0.004	UHPLC	USA	>4 weeks	Full breast expression	Not reported
Riboflavin	mg	Lima et al., 2020 (41)	13	0.0097	0.000064	UHPLC	USA	>4 weeks	Full breast expression	Not reported
Pantothenic acid	mg	Song et al., 1984 (42)	26	0.25	0.07	Radioimmunoassay	USA	12 weeks	Combined fore- and hindmilk	Morning
Vitamin B6	mg	Andon et al., 1989 (43)	30	0.012	0.0032	Microbiological method (*Saccharomyces Uvarum)*	USA	60 days	Foremilk	24 h period
Total Choline[1]	mg	Fischer et al., 2010 (44)	48	12.10	2.70	LC-MS	USA	45 days	Full breast expression	Morning
Total Water-Soluble Choline[2]	mg	Mokuarzel et al., 2019 (45)	20	17.45	2.74	LC-MS	Canada	2-6 months	Full breast expression	9-10 AM
	mg	Wiedman et al., 2018	301	11.17	3.09	LC-MS/MS	Canada	8 weeks	Hindmilk and full breast expression	Not reported
Folate	mcg	Panagos et al., 2016 (46)	21	4.50	4.09	*L. casei* microbial assay	USA	4-10 weeks	Full breast expression	Morning
	mcg	Houghton et al., 2009 (47)	23	8.32	2.78	*L. rhamnoses* microbiological assay	Canada	4-16 weeks	Full breast expression	1300 - 1450
	mcg	Mackey and Picciano, 1999 (48)	21	8.81	0.50	*L. casei* microbial assay	USA	3 and 6 months	Full breast expression	Not reported
	mcg	Udipi et al., 1987 (49)	27	8.12	1.73	*L. casei* microbial assay	USA	1 to 5 months	Mid-feed (after let-down)	12:00-1400 and 14-1600 as most representative of 24h

[1] Calculated as the sum of choline, phosphocholine, glycerophosphocholine, phosphatidylcholine, and sphingomyelin using a molecular weight for the choline moiety within each metabolite of 104.17 g/mol.
[2] Sum of free choline, phosphocholine, and glycerophosphocholine using a molecular weight for the choline moiety within each metabolite of 104.17 g/mol.

mcg	Smith et al., 1985 (50)	11	7.15	3.45	*L. casei* microbial assay	USA	6 and 12 weeks	Fore- and hind milk	Midday selected as most representative of 24h

E: Studies included in iHMNut 0-6 Months for Fat Soluble Vitamins

Nutrient	Reference	Sample Size	Mean	SD	Analytical Method	Location	Time post-partum	Milk Type	Time of Day
25-hydroxy Vitamin D (mcg)	Panagos et al., 2016 (46)	21	0.13	0.06	Radioimmunoassay	USA	4-10 weeks	Full breast expression	Morning
Vitamin K (mcg)	Ellis et al., 2022 (51)	23	0.13	0.02	LC-MS	USA	6 weeks	Full breast expression	Not reported
	Canfield et al., 1991 (52)	45	0.26	0.22	HPLC	USA	3 and 6 months	Full breast expression	24h
	Greer et al., 1997 (53)	11	0.11	0.04	HPLC	USA	6 and 12 weeks	Full breast expression	Not reported
Beta Carotene (mcg)	Lima et al., 2020 (41)	13	2.84	0.11	HPLC	USA	>4 weeks	Full breast expression	Not reported
Retinol (mcg)	Lima et al., 2020 (41)	13	64.99	5.82	HPLC	USA	>4 weeks	Full breast expression	Not reported
	Chappell et al., 1985 (54)	12	60.14	2.91	HPLC	Canada	37 days	Full breast expression	Early morning
	Panagos et al., 2016 (46)	21	4.46	1.65	HPLC	USA	4-10 weeks	Full breast expression	Morning
Vitamin E (mg)	Panagos et al., 2016 (46)	21	0.37	0.17	HPLC	USA	4-10 weeks	Full breast expression	

F: Studies included in iHMNut Fatty Acids and Cholesterol

**Reference**	**Smith et al., 2018** (55)	**Gaitan et al., 2018** (56)	**Clark et al, 1982** (15)	**Tijerina-Saenz et al., 2009** (57)	**Glew et al., 2008** (17)	**Glew et al., 2011** (18)
Sample size	13	24	10	60	29	19
Analytical method	GLC	LC-MS	GLC	GLC	GLC	GLC
Location	USA	USA	USA	Canada	USA	USA
Time post-partum	4 weeks	1 month	42-112 days	30 days	30-180	30-180 days
Milk Type	5-10 ml of breastmilk	Full expression from one breast	Full expression from one breast	Hind milk	First 3 ml was collected and discarded; the next 10-15 ml of milk was collected using a manual pump	Infant was offered one breast, and the contents of the other breast were expressed with an electrical pump
Time of Day	n/a	6-10 am	9:30-11:30 am and 1:30-3:30 pm	n/a	7 am - 10:30 am	8:30-10:30 am
SFA 4:0						0.000 (0.000)
SFA 6:0					0.0010 (0.001)	0.001 (0.001)
SFA 8:0					0.0030 (0.001)	0.004 (0.002)
SFA 10:0			0.043 (0.003)	0.026 (0.009)	0.0360 (0.009)	0.054 (0.014)
SFA 12:0			0.191 (0.023)	0.148 (0.056)	0.1400 (0.042)	0.250 (0.113)
SFA 14:0			0.256 (0.019)	0.188 (0.059)	0.1630 (0.050)	0.285 (0.116)
SFA 16:0			0.976 (0.089)	0.615 (0.089)	0.6150 (0.069)	1.004 (0.105)
SFA 18:0			0.342 (0.062)	0.194 (0.047)	0.2080 (0.039)	0.340 (0.043)
MUFA 16:1			0.162 (0.012)	0.082 (0.021)	0.0720 (0.019)	
MUFA 18:1			1.685 (0.256)	1.070 (0.131)	1.0600 (0.111)	1.452 (0.163)
MUFA 20:1					0.0120 (0.003)	

MUFA 22:1					0.0030 (0.001)	0.003 (0.003)
LA			0.680 (0.080)	0.406 (0.096)	0.6510 (0.150)	0.834 (0.151)
ALA			0.068 (0.009)	0.045 (0.016)	0.0440 (0.001)	0.061 (0.024)
PUFA 20:3			0.014 (0.001)		0.0120 (0.003)	0.019 (0.016)
ARA			0.019 (0.001)	0.013 (0.002)	0.0140 (0.003)	0.014 (0.004)
EPA		1.322E-04 (9.052 E-05)		0.003 (0.002)	0.003 (0.001)	0.002 (0.004)
PUFA 22:4n6					0.0030 (0.001)	0.004 (0.002)
PUFA 22:5n-3 (DPA)					0.0050 (0.000)	0.003 (0.001)
DHA	0.015 (0.0124)	0.0002 (0.0001)		0.009 (0.005)	0.0040 (0.001)	0.005 (0.002)
Cholesterol			9.83 (0.37)			

Supplemental Table 3: Studies from NASEM scanning review used in the comparison weighted means and pooled standard deviations cited in Table 2[1]

Component	References for comparable studies from NASEM Scanning Review – see (1)	Studies Identified in NASEM Scanning Review Not Included in Weighted NASEM Average for Comparison
Protein	Carratù et al., 2003 (58), McCrory et al., 1999 (14), Dewey et al., 1994 (24), Stuff and Nichols, 1989 (12)	
Total Lipid	Wong et al., 2019 (59); Martysiak-Zurowska et al., 2013 (60); Szlagatys-Sidorkiewicz et al., 2013 (61); Clark et al., 1989 (62); Ferris et al., 1988 (7); Nommsen et al., 1991 (3); McCrory et al., 1999 (14); Dewey et al., 1994 (24); Stuff and Nichols, 1989 (12); Butte et al., 1984 (10)	
Lactose	Goran et al., 2017 (19); Perrin et al., 2017 (63); Zimmerman et al., 2009 (64)., Wack et al., 1997 (22); Huisman et al., 1996 (65); Coppa et al., 1993 (66); Jozwik et al., 2013 (67)	
Iron	Taravati et al., 2018 (68); Mahdavi et al., 2010 (69); Mahdavi et al., 2015 (69); Hannan et al., 2009 (25); Domellöf et al., 2004 (70); Silvestre et al., 2001 (71); Bocca et al., 2000 (72), Butte et al., 1987 (27); Dewey and Lönnerdal, 1983 (73); Dewey et al., 1984 (24); Feeley et al., 1983 (23); Picciano et al., 1981 (26)	
Zinc	Taravati et al., 2018 (68); Mahdavi et al., 2010 (69); Mahdavi et al., 2015 (74); Hannan et al., 2009 (25); Domellöf et al., 2004 (70); Silvestre et al., 2001 (71); Bocca et al., 2000 (72); Butte et al., 1987 (27); Dewey and Lönnerdal, 1983 (73); Dewey et al., 1984 (24); Feeley et al., 1983 (23); Picciano et al., 1981 (26); Qian et al., 2012 (75); Doneray et al., 2017 (76); Severi et al., 2013 (77); Hunt et al., 2005 (78); Kantola and Vartiainen, 2001 (79); Wasowicz et al., 2001 (80); Lin et al., 1998 (81); Ortega et al., 1997 (82); Ohtake et al., 1981 (83); Dagnelie et al., 1992 (84); Casey et al., 1989 (30); Butte et al., 1984 (6); Ohtake and Tamura., 1993 (85); Krebs et al., 1985 (33); Casey et al., 1985 (31); Matos et al., 2009 (86); Krebs et al., 1985 (34)	Dumrongwongsiri et al., 2015 (87) – data unavailable to calculate variance.<br><br>Krebs et al., 1994 (88) – human milk concentrations not reported, just daily intakes of zinc from human milk.
Copper	Taravati et al., 2018 (68); Mahdavi et al., 2010 (69); Mahdavi et al., 2015 (74); Domellöf et al., 2004 (70); Silvestre et al., 2001 (71); Bocca et al., 2000 (72), Butte et al., 1987 (27); Dewey and Lönnerdal, 1983 (73); Dewey et al., 1984 (24); Feeley et al., 1983 (23); Picciano et al., 1981 (26); Kantola and Vartiainen, 2001 (79); Wasowicz et al., 2001 (80); Lin et al., 1998 (81); Ohtake and Tamura., 1993 (85); Ohtake et al., 1981 (83); Casey et al., 1989 (30); Casey et al., 1985 (31); Matos et al., 2009 (86);	
Selenium	Matos et al., 2009 (86); Kantola and Vartiainen, 2001 (79); Li et al., 1999 (89); Tamari and Kim, 1999 (90); Rodríguez Rodríguez et al., 1998 (91)	
Manganese	Bocca et al., 2000 (72); Matos et al., 2009 (86); Qian et al., 2010 (92); Casey et al., 1989 (30); Casey et al., 1985 (31);	
Magnesium	Taravati et al., 2018 (68); Mahdavi et al., 2015 (74); Bocca et al., 2000 (72); Butte et al., 1987 (27); Dewey et al., 1984 (24); Picciano et al., 1981 (26); Dewey and Lönnerdal, 1983 (73); Butts et al., 2018 (93); Kim et al., 2017 (94); Zhao et al. 2014 (95); Hunt et al., 2005 (78); Urzica et al., 2013 (96); Lin et al., 1998 (81); Dagnelie et al., 1992 (84); Butte et al., 1984 (6); Fly et al., 1998 (97); Feeley et al., 1983 (36); Greer et al., 1982 (98)	

Iodine	Wang et al., 2018 (99); Wack et al., 1997 (100);	Dold et al., 2017 (101) excluded because units (mcg/day) could not be converted to concentration per g of human milk.
Calcium	Taravati et al., 2018 (68); Mahdavi et al., 2015 (74); Bocca et al., 2000 (72), Butte et al., 1987 (27); Dewey and Lönnerdal, 1983 (73); Dewey et al., 1984 (24); Picciano et al., 1981 (26); Minato et al., 2019 (102); Butts et al., 2018 (93); Kim et al., 2017 (94); Zhao et al. 2014 (95); Perrin et al., 2017 (63); Qian et al., 2010 (92); Hunt et al., 2005 (78); Lin et al., 1998 (81); Dagnelie et al., 1992 (84); Butte et al., 1984 (6); Fly et al., 1998 (97); Feeley et al., 1983 (36); Greer et al., 1982 (98); Shehadeh et al., 2006 (103); Mataloun and Leone., 2000 (104)	
Potassium	Kim et al., 2017 (94); Zhao et al. 2014 (95); Perrin et al., 2017 (63); Qian et al., 2010 (92); Fly et al., 1998 (97); Wack et al., 1997 (100); Butte et al., 1987 (27); Morris et al., 1986 (105); Dewey and Lönnerdal, 1983 (73); Dewey et al., 1984 (24); Picciano et al., 1981 (26); Keenan et al., 1992 (39);	
Sodium	Kim et al., 2017 (94); Zhao et al. 2014 (95); Perrin et al., 2017 (63); Qian et al., 2010 (92); Koo and Gupta., 1982 (106); Taravati et al., 2018 (68); Zimmerman et al., 2009 (64); Shehadeh et al., 2006 (103); Fly et al., 1998 (97); Wack et al., 1997 (100); Butte et al., 1987 (27); Morris et al., 1986 (105); Butte et al., 1984 (6); Dewey and Lönnerdal, 1983 (73); Dewey et al., 1984 (24); Picciano et al., 1981 (26); Keenan et al., 1992 (39);	
Phosphorous	Picciano et al., 1981 (26); Minato et al., 2019 (102); Kim et al., 2017 (94); Zhao et al. 2014 (95); Qian et al., 2010 (92); Feeley et al., 1983 (36); Mataloun and Leone., 2000 (104); Morris et al., 1986 (105);	
Chloride	Wack et al., 1997 (100); Morris et al., 1986 (105); Picciano et al., 1981 (26);	
Thiamin	Sunaric et al., 2017 (107)	
Riboflavin	Sunaric et al., 2017 (107)	
Vitamin K	Greer et al., 1997 (53); Canfield et al., 1991 (108)	
Vitamin A	Denic et al., 2019 (109); Panagos et al., 2016 (46); Szlagatys-Sidorkiewicz et al., 2012 (110); Meneses and Trugo, 2005 (111); Schweigert et al., 2004 (112); Canfield et al., 2003 (113); Gossage et al., 2002 (114); Chappell et al., 1985 (115)	

[1] Concentration data from studies extracted in the NASEM scanning review were converted into standard units, and summarized as weighted means and pooled standard deviations as described in the Materials and Methods section to provide a point of comparison with iHMNut values. Where multiple values were provided for a study (e.g for multiple timepoints or regions), the weighted mean of all values was used. Data for individual studies available in the reference (1)

References

1. National Academies of Sciences, Engineering, and Medicine. Scanning for New Evidence on the Nutrient Content of Human Milk: A Process Model for Determining Age-Specific Nutrient Requirements. Washington, DC: The National Academies Press, 2020.

2. Mohr AE, Senkus KE, McDermid JM, Berger PK, Perrin MT, Handu D. Human Milk Nutrient Composition Data is Critically Lacking in the United States and Canada: Results from a Systematic Scoping Review of 2017-2022. Adv Nutr 2023;S2161–7.

3. Nommsen LA, Lovelady CA, Heinig MJ, Lönnerdal B, Dewey KG. Determinants of energy, protein, lipid, and lactose concentrations in human milk during the first 12 mo of lactation: the DARLING Study. Am J Clin Nutr 1991;53:457–65.

4. Butte NF, Wong WW, Ferlic L, Smith EO, Klein PD, Garza C. Energy expenditure and deposition of breast-fed and formula-fed infants during early infancy. Pediatr Res 1990;28:631–40.

5. Gross SJ, David RJ, Bauman L, Tomarelli RM. Nutritional composition of milk produced by mothers delivering preterm. J Pediatr 1980;96:641–4.

6. Butte NF, Garza C, Johnson CA, Smith EO, Nichols BL. Longitudinal changes in milk composition of mothers delivering preterm and term infants. Early Hum Dev 1984;9:153–62.

7. Ferris AM, Dotts MA, Clark RM, Ezrin M, Jensen RG. Macronutrients in human milk at 2, 12, and 16 weeks postpartum. J Am Diet Assoc 1988;88:694–7.

8. Garza C, Butte NF. Energy concentration of human milk estimated from 24-h pools and various abbreviated sampling schemes. J Pediatr Gastroenterol Nutr 1986;5:943–8.

9. Dewey KG, Lönnerdal B. Milk and nutrient intake of breast-fed infants from 1 to 6 months: relation to growth and fatness. J Pediatr Gastroenterol Nutr 1983;2:497–506.

10. Butte NF, Garza C, Smith EO, Nichols BL. Human milk intake and growth in exclusively breast-fed infants. J Pediatr 1984;104:187–95.

11. Garza C, Johnson CA, Smith EO, Nichols BL. Changes in the nutrient composition of human milk during gradual weaning. Am J Clin Nutr 1983;37:61–5.

12. Stuff JE, Nichols BL. Nutrient intake and growth performance of older infants fed human milk. J Pediatr 1989;115:959–68.

13. Dewey KG, Lovelady CA, Nommsen-Rivers LA, McCrory MA, Lönnerdal B. A randomized study of the effects of aerobic exercise by lactating women on breast-milk volume and composition. N Engl J Med 1994;330:449–53.

14. McCrory MA, Nommsen-Rivers LA, Molé PA, Lönnerdal B, Dewey KG. Randomized trial of the short-term effects of dieting compared with dieting plus aerobic exercise on lactation performance. Am J Clin Nutr 1999;69:959–67.

15. Clark RM, Ferris AM, Fey M, Brown PB, Hundrieser KE, Jensen RG. Changes in the lipids of human milk from 2 to 16 weeks postpartum. J Pediatr Gastroenterol Nutr 1982;1:311–5.

16. Clark RM, Fey MB, Jensen RG, Hill DW. Desmosterol in human milk. Lipids 1983;18:264–6.

17. Glew RH, Wold RS, Corl B, Calvin CD, Vanderjagt DJ. Low docosahexaenoic acid in the diet and milk of American Indian women in New Mexico. J Am Diet Assoc 2011;111:744–8.

18. Glew RH, Wold RS, Corl B, Calvin CD, Vanderjagt DJ. Low docosahexaenoic acid in the diet and milk of American Indian women in New Mexico. J Am Diet Assoc 2011;111:744–8.

19. Goran MI, Martin AA, Alderete TL, Fujiwara H, Fields DA. Fructose in Breast Milk Is Positively Associated with Infant Body Composition at 6 Months of Age. Nutrients 2017;9:146.

20. Berger PK, Fields DA, Demerath EW, Fujiwara H, Goran MI. High-Fructose Corn-Syrup-Sweetened Beverage Intake Increases 5-Hour Breast Milk Fructose Concentrations in Lactating Women. Nutrients 2018;10:669.

21. Nijman RM, Liu Y, Bunyatratchata A, Smilowitz JT, Stahl B, Barile D. Characterization and Quantification of Oligosaccharides in Human Milk and Infant Formula. J Agric Food Chem 2018;66:6851–9.

22. Wack RP, Lien EL, Taft D, Roscelli JD. Electrolyte composition of human breast milk beyond the early postpartum period. Nutrition 1997;13:774–7.

23. Feeley RM, Eitenmiller RR, Jones JB, Barnhart H. Copper, iron, and zinc contents of human milk at early stages of lactation. Am J Clin Nutr 1983;37:443–8.

24. Dewey KG, Finley DA, Lönnerdal B. Breast milk volume and composition during late lactation (7-20 months). J Pediatr Gastroenterol Nutr 1984;3:713–20.

25. Hannan MA, Faraji B, Tanguma J, Longoria N, Rodriguez RC. Maternal milk concentration of zinc, iron, selenium, and iodine and its relationship to dietary intakes. Biol Trace Elem Res 2009;127:6–15.

26. Picciano MF, Calkins EJ, Garrick JR, Deering RH. Milk and mineral intakes of breastfed infants. Acta Paediatr Scand 1981;70:189–94.

27. Butte NF, Garza C, Smith EO, Wills C, Nichols BL. Macro- and trace-mineral intakes of exclusively breast-fed infants. Am J Clin Nutr 1987;45:42–8.

28. Friel JK, Andrews WL, Jackson SE, Longerich HP, Mercer C, McDonald A, Dawson B, Sutradhar B. Elemental composition of human milk from mothers of premature and full-term infants during the first 3 months of lactation. Biol Trace Elem Res 1999;67:225–47.

29. Hopperton KE, O'Neill E, Chakrabarti S, Stanton M, Parnel S, Arbuckle TE, Ashley-Martin J, Bertinato J, Bouchard MF, Borghese MM, et al. Concentrations and predictors of select nutrients in Canadian human milk samples from the Maternal-Infant Research on Environmental Chemicals pregnancy cohort. Am J Clin Nutr 2024;S0002–0.

30. Casey CE, Neville MC, Hambidge KM. Studies in human lactation: secretion of zinc, copper, and manganese in human milk. Am J Clin Nutr 1989;49:773–85.

31. Casey CE, Hambidge KM, Neville MC. Studies in human lactation: zinc, copper, manganese and chromium in human milk in the first month of lactation. Am J Clin Nutr 1985;41:1193–200.

32. Hunt CD, Butte NF, Johnson LK. Boron concentrations in milk from mothers of exclusively breast-fed healthy full-term infants are stable during the first four months of lactation. J Nutr 2005;135:2383–6.

33. Krebs NF, Hambidge KM, Jacobs MA, Rasbach JO. The effects of a dietary zinc supplement during lactation on longitudinal changes in maternal zinc status and milk zinc concentrations. Am J Clin Nutr 1985;41:560–70.

34. Krebs NF, Hambidge KM, Jacobs MA, Mylet S. Zinc in human milk: diurnal and within-feed patterns. J Pediatr Gastroenterol Nutr 1985;4:227–9.

35. Bertinato J, Gaudet J, De Silva N, Mohanty S, Qiao C, Herod M, Gharibeh N, Weiler H. Iodine Status of Mother-Infant Dyads from Montréal, Canada: Secondary Analyses of a Vitamin D Supplementation Trial in Breastfed Infants. J Nutr 2022;152:1459–66.

36. Feeley RM, Eitenmiller RR, Jones JB, Barnhart H. Calcium, phosphorus, and magnesium contents of human milk during early lactation. J Pediatr Gastroenterol Nutr 1983;2:262–7.

37. Fly AD, Uhlin KL, Wallace JP. Major mineral concentrations in human milk do not change after maximal exercise testing. Am J Clin Nutr 1998;68:345–9.

38. Morriss FH, Brewer ED, Spedale SB, Riddle L, Temple DM, Caprioli RM, West MS. Relationship of human milk pH during course of lactation to concentrations of citrate and fatty acids. Pediatrics 1986;78:458–64.

39. Keenan BS, Buzek SW, Garza C, Potts E, Nichols BL. Diurnal and longitudinal variations in human milk sodium and potassium: implication for nutrition and physiology. Am J Clin Nutr 1982;35:527–34.

40. Allen JC, Keller RP, Archer P, Neville MC. Studies in human lactation: milk composition and daily secretion rates of macronutrients in the first year of lactation. Am J Clin Nutr 1991;54:69–80.

41. Lima HK, Vogel K, Hampel D, Wagner-Gillespie M, Fogleman AD. The Associations Between Light Exposure During Pumping and Holder Pasteurization and the Macronutrient and Vitamin Concentrations in Human Milk. J Hum Lact 2020;36:254–63.

42. Song WO, Chan GM, Wyse BW, Hansen RG. Effect of pantothenic acid status on the content of the vitamin in human milk. Am J Clin Nutr 1984;40:317–24.

43. Andon MB, Reynolds RD, Moser-Veillon PB, Howard MP. Dietary intake of total and glycosylated vitamin B-6 and the vitamin B-6 nutritional status of unsupplemented lactating women and their infants. Am J Clin Nutr 1989;50:1050–8.

44. Fischer LM, da Costa KA, Galanko J, Sha W, Stephenson B, Vick J, Zeisel SH. Choline intake and genetic polymorphisms influence choline metabolite concentrations in human breast milk and plasma. Am J Clin Nutr 2010;92:336–46.

45. Moukarzel S, Wiedeman AM, Soberanes LS, Dyer RA, Innis SM, Lamers Y. Variability of Water-Soluble Forms of Choline Concentrations in Human Milk during Storage, after Pasteurization, and among Women. Nutrients 2019;11:3024.

46. Panagos P, Vishwanathan GR, Penfield-Cyr A, Matthan NR, Shivappa N, Wirth MD, Hebert JR, Sen S. Breastmilk from obese mothers has pro-inflammatory properties and decreased neuroprotective factors. Journal of Perinatology 2016;36:284–90.

47. Houghton LA, Yang J, O'Connor DL. Unmetabolized folic acid and total folate concentrations in breast milk are unaffected by low-dose folate supplements. Am J Clin Nutr 2009;89:216–20.

48. Mackey AD, Picciano MF. Maternal folate status during extended lactation and the effect of supplemental folic acid. Am J Clin Nutr 1999;69:285–92.

49. Udipi SA, Kirksey A, Roepke JL. Diurnal variations in folacin levels of human milk: use of a single sample to represent folacin concentration in milk during a 24-h period. Am J Clin Nutr 1987;45:770–9.

50. Smith AM, Picciano MF, Deering RH. Folate supplementation during lactation: maternal folate status, human milk folate content, and their relationship to infant folate status. J Pediatr Gastroenterol Nutr 1983;2:622–8.

51. Ellis JL, Wang M, Fu X, Fields CJ, Donovan SM, Booth SL. Feeding Practice and Delivery Mode Are Determinants of Vitamin K in the Infant Gut: An Exploratory Analysis. Curr Dev Nutr 2022;6:nzac019.

52. Canfield LM, Hopkinson JM, Lima AF, Silva B, Garza C. Vitamin K in colostrum and mature human milk over the lactation period--a cross-sectional study. Am J Clin Nutr 1991;53:730–5.

53. Greer FR, Marshall SP, Foley AL, Suttie JW. Improving the vitamin K status of breastfeeding infants with maternal vitamin K supplements. Pediatrics 1997;99:88–92.

54. Chappell JE, Francis T, Clandinin MT. Vitamin A and E content of human milk at early stages of lactation. Early Hum Dev 1985;11:157–67.

55. Smith S, Kevala K, Cunningham B, Rouse C, Hunt CE, Kim H. N-docosahexaenoylethanolamine detected in human breast milk. Prostaglandins Leukot Essent Fatty Acids 2018;137:1–4.

56. Gaitán AV, Wood JT, Zhang F, Makriyannis A, Lammi-Keefe CJ. Endocannabinoid Metabolome Characterization of Transitional and Mature Human Milk. Nutrients 2018;10:1294.

57. Tijerina-Sáenz A, Innis SM, Kitts DD. Antioxidant capacity of human milk and its association with vitamins A and E and fatty acid composition. Acta Paediatr 2009;98:1793–8.

58. Carratù B, Boniglia C, Scalise F, Ambruzzi AM, Sanzini E. Nitrogenous components of human milk: non-protein nitrogen, true protein and free amino acids. Food Chemistry 2003;81:357–62.

59. Wong VW, Ng Y, Chan S, Su Y, Kwok KW, Chan H, Cheung C, Lee H, Pak W, Li S, et al. Positive relationship between consumption of specific fish type and n-3 PUFA in milk of Hong Kong lactating mothers. Br J Nutr 2019;121:1431–40.

60. Martysiak-Żurowska D, Szlagatys-Sidorkiewicz A, Zagierski M. Concentrations of alpha- and gamma-tocopherols in human breast milk during the first months of lactation and in infant formulas. Matern Child Nutr 2013;9:473–82.

61. Szlagatys-Sidorkiewicz A, Martysiak-Żurowska D, Krzykowski G, Zagierski M, Kamińska B. Maternal smoking modulates fatty acid profile of breast milk. Acta Paediatr 2013;102:353.

62. Clark RM, Hundrieser KE. Changes in cholesteryl esters of human milk with total milk lipid. J Pediatr Gastroenterol Nutr 1989;9:347–50.

63. Perrin MT, Fogleman AD, Newburg DS, Allen JC. A longitudinal study of human milk composition in the second year postpartum: implications for human milk banking. Matern Child Nutr 2017;13:e12239.

64. Zimmerman DR, Goldstein L, Lahat E, Braunstein R, Stahi D, Bar-Haim A, Berkovitch M. Effect of a 24+ hour fast on breast milk composition. J Hum Lact 2009;25:194–8.

65. Huisman M, van Beusekom CM, Lanting CI, Nijeboer HJ, Muskiet FA, Boersma ER. Triglycerides, fatty acids, sterols, mono- and disaccharides and sugar alcohols in human milk and current types of infant formula milk. Eur J Clin Nutr 1996;50:255–60.

66. Coppa GV, Gabrielli O, Pierani P, Catassi C, Carlucci A, Giorgi PL. Changes in carbohydrate composition in human milk over 4 months of lactation. Pediatrics 1993;91:637–41.

67. Jóźwik M, Jóźwik M, Teng C, Jóźwik M, Battaglia FC. Human breast milk sugars and polyols over the first 10 puerperium days. Am J Hum Biol 2013;25:198–204.

68. Taravati Javad M, Vahidinia A, Samiee F, Elaridi J, Leili M, Faradmal J, Rahmani A. Analysis of aluminum, minerals and trace elements in the milk samples from lactating mothers in Hamadan, Iran. J Trace Elem Med Biol 2018;50:8–15.

69. Mahdavi R, Nikniaz L, Gayemmagami SJ. Association between zinc, copper, and iron concentrations in breast milk and growth of healthy infants in Tabriz, Iran. Biol Trace Elem Res 2010;135:174–81.

70. Domellöf M, Lönnerdal B, Dewey KG, Cohen RJ, Hernell O. Iron, zinc, and copper concentrations in breast milk are independent of maternal mineral status. Am J Clin Nutr 2004;79:111–5.

71. Silvestre D, Martìnez-Costa C, Lagarda MJ, Brines J, Farré R, Clemente G. Copper, iron, and zinc contents in human milk during the first three months of lactation: a longitudinal study. Biol Trace Elem Res 2001;80:1–11.

72. Bocca B, Alimonti A, Coni E, Di Pasquale M, Giglio L, Bocca AP, Caroli S. Determination of the total content and binding pattern of elements in human milk by highperformance liquid chromatography-inductively coupled plasma atomic emission spectrometry. Talanta 2000;53:295–303.

73. Dewey KG, Lönnerdal B. Milk and nutrient intake of breast-fed infants from 1 to 6 months: relation to growth and fatness. J Pediatr Gastroenterol Nutr 1983;2:497–506.

74. Mahdavi R, Taghipour S, Ostadrahimi A, Nikniaz L, Hezaveh SJG. A pilot study of synbiotic supplementation on breast milk mineral concentrations and growth of exclusively breast fed infants. J Trace Elem Med Biol 2015;30:25–9.

75. Qian L, Wang B, Tang N, Zhang W, Cai W. Polymorphisms of SLC30A2 and selected perinatal factors associated with low milk zinc in Chinese breastfeeding women. Early Hum Dev 2012;88:663–8.

76. Doneray H, Olcaysu E, Yildirim A, Ozden A. The effect of the zinc concentration in breast milk on neonatal weight gain. J Trace Elem Med Biol 2017;41:32–5.

77. Severi C, Hambidge M, Krebs N, Alonso R, Atalah E. Zinc in plasma and breast milk in adolescents and adults in pregnancy and pospartum: a cohort study in Uruguay. Nutr Hosp 2013;28:223–8.

78. Hunt CD, Butte NF, Johnson LK. Boron concentrations in milk from mothers of exclusively breast-fed healthy full-term infants are stable during the first four months of lactation. J Nutr 2005;135:2383–6.

79. Kantola M, Vartiainen T. Changes in selenium, zinc, copper and cadmium contents in human milk during the time when selenium has been supplemented to fertilizers in Finland. Journal of Trace Elements in Medicine & Biology 2001;15:11–7.

80. Wasowicz W, Gromadzinska J, Szram K, Rydzynski K, Cieslak J, Pietrzak Z. Selenium, zinc, and copper concentrations in the blood and milk of lactating women. Biol Trace Elem Res 2001;79:221–33.

81. Lin TH, Jong YJ, Chiang CH, Yang MH. Longitudinal changes in Ca, Mg, Fe, Cu, and Zn in breast milk of women in Taiwan over a lactation period of one year. Biol Trace Elem Res 1998;62:31–41.

82. Ortega RM, Andrés P, Martínez RM, López-Sobaler AM, Quintas ME. Zinc levels in maternal milk: the influence of nutritional status with respect to zinc during the third trimester of pregnancy. Eur J Clin Nutr 1997;51:253–8.

83. Ohtake M, Chiba R, Mochizuki K, Tada K. Zinc and copper concentrations in human milk and in serum from exclusively-breast-fed infants during the first 3 months of life. Tohoku J Exp Med 1981;135:335–43.

84. Dagnelie PC, van Staveren WA, Roos AH, Tuinstra LG, Burema J. Nutrients and contaminants in human milk from mothers on macrobiotic and omnivorous diets. Eur J Clin Nutr 1992;46:355–66.

85. Ohtake M, Tamura T. Changes in zinc and copper concentrations in breast milk and blood of Japanese women during lactation. J Nutr Sci Vitaminol (Tokyo) 1993;39:189–200.

86. Matos C, Moutinho C, Balcao V, Almeida C, Ribeiro M, Marques AF, Guerra A. Total antioxidant activity and trace elements in human milk: The first 4 months of breast-feeding. European Food Research and Technology 2009;230:547–51.

87. Dumrongwongsiri O, Suthutvoravut U, Chatvutinun S, Phoonlabdacha P, Sangcakul A, Siripinyanond A, Thiengmanee U, Chongviriyaphan N. Maternal zinc status is associated with breast milk zinc concentration and zinc status in breastfed infants aged 4-6 months. Asia Pac J Clin Nutr 2015;24:273–80.

88. Krebs NF, Reidinger CJ, Robertson AD, Hambidge KM. Growth and intakes of energy and zinc in infants fed human milk. J Pediatr 1994;124:32–9.

89. Li F, Rossipal E, Irgolic KJ. Determination of selenium in human milk by hydride cold-trapping atomic absorption spectrometry and calculation of daily selenium intake. J Agric Food Chem 1999;47:3265–8.

90. Tamari Y, Kim ES. Longitudinal study of the dietary selenium intake of exclusively breast-fed infants during early lactation in Korea and Japan. J Trace Elem Med Biol 1999;13:129–33.

91. Rodríguez Rodríguez EM, Sanz Alaejos M, Díaz Romero C. Concentrations of selenium in human milk. Z Lebensm Unters Forsch 1998;207:174–9.

92. Qian J, Chen T, Lu W, Wu S, Zhu J. Breast milk macro- and micronutrient composition in lactating mothers from suburban and urban Shanghai. J Paediatr Child Health 2010;46:115–20.

93. Butts CA, Hedderley DI, Herath TD, Paturi G, Glyn-Jones S, Wiens F, Stahl B, Gopal P. Human Milk Composition and Dietary Intakes of Breastfeeding Women of Different Ethnicity from the Manawatu-Wanganui Region of New Zealand. Nutrients 2018;10:1231.

94. Kim H, Jung B-, Lee B-, Kim YJ, Jung JA, Chang N. Retinol, α-tocopherol, and selected minerals in breast milk of lactating women with full-term infants in South Korea. Nutrition Research and Practice 2017;11:64–9.

95. Zhao A, Ning Y, Zhang Y, Yang X, Wang J, Li W, Wang P. Mineral compositions in breast milk of healthy Chinese lactating women in urban areas and its associated factors. Chin Med J (Engl) 2014;127:2643–8.

96. Urzica D, Gales C, Zamfir C, Nechifor M. The influence of oral steroidal contraceptives on magnesium concentration in breast milk. Magnes Res 2013;26:188–91.

97. Fly AD, Uhlin KL, Wallace JP. Major mineral concentrations in human milk do not change after maximal exercise testing. Am J Clin Nutr 1998;68:345–9.

98. Greer FR, Tsang RC, Levin RS, Searcy JE, Wu R, Steichen JJ. Increasing serum calcium and magnesium concentrations in breast-fed infants: Longitudinal studies of minerals in human milk and in sera of nursing mothers and their infants. J Pediatr 1982;100:59–64.

99. Wang W, Sun Y, Zhang M, Zhang Y, Chen W, Tan L, Shen J, Zhao Z, Lan S, Zhang W. Breast milk and infant iodine status during the first 12 weeks of lactation in Tianjin City, China. Asia Pac J Clin Nutr 2018;27:393–8.

100. Wack RP, Lien EL, Taft D, Roscelli JD. Electrolyte composition of human breast milk beyond the early postpartum period. Nutrition 1997;13:774–7.

101. Dold S, Zimmermann MB, Aboussad A, Cherkaoui M, Jia Q. Breast milk iodine concentration is a more accurate biomarker of iodine status than urinary iodine concentration in exclusively breastfeeding women. Journal of Nutrition 2017;147:528–37.

102. Minato T, Nomura K, Asakura H, Aihara A, Hiraike H, Hino Y, Isojima T, Kodama H. Maternal Undernutrition and Breast Milk Macronutrient Content Are Not Associated with Weight in Breastfed Infants at 1 and 3 Months after Delivery. Int J Environ Res Public Health 2019;16:3315.

103. Shehadeh N, Aslih N, Shihab S, Werman MJ, Sheinman R, Shamir R. Human milk beyond one year post-partum: lower content of protein, calcium, and saturated very long-chain fatty acids. J Pediatr 2006;148:122–4.

104. Mataloun MM, Leone CR. Human milk mineral intake and serum concentrations of calcium and phosphorus in newborn term infants: influence of intrauterine growth restriction. Acta Paediatr 2000;89:1093–7.

105. Morriss FH, Brewer ED, Spedale SB, Riddle L, Temple DM, Caprioli RM, West MS. Relationship of human milk pH during course of lactation to concentrations of citrate and fatty acids. Pediatrics 1986;78:458–64.

106. Koo WW, Gupta JM. Breast milk sodium. Arch Dis Child 1982;57:500–2.

107. Sunarić S, Denić M, Lalić J, Jovanović T, Spasić A, Živković J, Trutić N, Kocić G. Physicochemical and biochemical parameters in milk of Serbian breastfeeding women. Turk J Med Sci 2017;47:246–51.

108. Canfield LM, Hopkinson JM, Lima AF, Silva B, Garza C. Vitamin K in colostrum and mature human milk over the lactation period--a cross-sectional study. Am J Clin Nutr 1991;53:730–5.

109. Denić M, Sunarić S, Genčić M, Živković J, Jovanović T, Kocić G, Jonović M. Maternal age has more pronounced effect on breast milk retinol and β-carotene content than maternal dietary pattern. Nutrition 2019;65:120–5.

110. Szlagatys-Sidorkiewicz A, Zagierski M, Jankowska A, Łuczak G, Macur K, Bączek T, Korzon M, Krzykowski G, Martysiak-Żurowska D, Kamińska B. Longitudinal study of vitamins A, E and lipid oxidative damage in human milk throughout lactation. Early Hum Dev 2012;88:421–4.

111. Meneses F, Trugo NMF. Retinol, *β*-carotene, and lutein + zeaxanthin in the milk of Brazilian nursing women: associations with plasma concentrations and influences of maternal characteristics. Nutrition Research 2005;25:443–51.

112. Schweigert FJ, Bathe K, Chen F, Büscher U, Dudenhausen JW. Effect of the stage of lactation in humans on carotenoid levels in milk, blood plasma and plasma lipoprotein fractions. Eur J Nutr 2004;43:39–44.

113. Canfield LM, Clandinin MT, Davies DP, Fernandez MC, Jackson J, Hawkes J, Goldman WJ, Pramuk K, Reyes H, Sablan B, et al. Multinational study of major breast milk carotenoids of healthy mothers. Eur J Nutr 2003;42:133–41.

114. Gossage CP, Deyhim M, Yamini S, Douglass LW, Moser-Veillon PB. Carotenoid composition of human milk during the first month postpartum and the response to beta-carotene supplementation. Am J Clin Nutr 2002;76:193–7.

115. Chappell JE, Francis T, Clandinin MT. Vitamin A and E content of human milk at early stages of lactation. Early Hum Dev 1985;11:157–67.
